# Bandwagoning, free‐riding and heterogeneity in influenza vaccine decisions: An online experiment

**DOI:** 10.1002/hec.4467

**Published:** 2022-01-06

**Authors:** Matteo M. Galizzi, Krystal W. Lau, Marisa Miraldo, Katharina Hauck

**Affiliations:** ^1^ Department of Psychological and Behavioral Science LSE Behavioral Science Hub LSE Global Health Initiative London School of Economics London UK; ^2^ Department of Economics and Public Policy Centre for Health Economics & Policy Innovation Imperial College Business School London UK; ^3^ MRC Centre for Global Infectious Disease Analysis Jameel Institute for Disease and Emergency Analytics School of Public Health, Imperial College London London UK

**Keywords:** behavioral economics, free‐riding, influenza, online experiments, social norms, vaccines

## Abstract

‘Nudge’‐based social norms messages conveying high population influenza vaccination coverage levels can encourage vaccination due to bandwagoning effects but also discourage vaccination due to free‐riding effects on low risk of infection, making their impact on vaccination uptake ambiguous. We develop a theoretical framework to capture heterogeneity around vaccination behaviors, and empirically measure the causal effects of different messages about vaccination coverage rates on four self‐reported and behavioral vaccination intention measures. In an online experiment, *N* = 1365 UK adults are randomly assigned to one of seven treatment groups with different messages about their social environment's coverage rate (varied between 10% and 95%), or a control group with no message. We find that treated groups have significantly greater vaccination intention than the control. Treatment effects increase with the coverage rate up to a 75% level, consistent with a bandwagoning effect. For coverage rates above 75%, the treatment effects, albeit still positive, stop increasing and remain flat (or even decline). Our results suggest that, at higher coverage rates, *free‐riding* behavior may partially crowd out *bandwagoning* effects of coverage rate messages. We also find significant heterogeneity of these effects depending on the individual perceptions of risks of infection and of the coverage rates.

## 1 INTRODUCTION

The objective of our study is to experimentally evaluate the impact of social norms messages about vaccination coverage rates on individuals' seasonal influenza vaccination intentions. Seasonal influenza poses a severe public health and economic burden to society, with 3–5 million new cases and 290,000–650,000 deaths annually worldwide (World Health Organization, 2018), costing an estimated $11.2 billion each year in the United States (Putri et al., 2018). Although vaccines have been shown to be a low‐cost yet highly effective method of reducing disease burden (Quinn et al., 2017), vaccination rates remain suboptimal (Jorgensen et al., 2018).

Many health governmental organizations have set target population influenza vaccination coverage rates. The World Health Organization and the European Council recommend that 75% of elderly (65 years‐old and over) and at‐risk individuals (including those with chronic illness, pregnant women, and health‐care workers) receive the flu vaccine (European Union Council, 2009; World Health Assembly, 2003). Public Health England aims to vaccinate 75% of elderly, 55% of at‐risk adults (18–64 years‐old), and 50%–65% of children (2–10 years‐old) against the flu in the U.K. (Public Health England, 2019). The U.S. Department of Health and Human Services set the most ambitious targets to vaccinate 90% of elderly, 80%–90% of all adults aged 18–64 years, and children aged 18 years and under in the U.S. (Office of Disease Prevention and Health Promotion, 2019).

Although some of these targets were set over 15 years ago, many countries still fall short today. During the 2017–18 influenza season, only 37.1% of U.S. adults aged 18 and over and 59.6% of elderly aged 65 and over got vaccinated (Centers for Disease Control and Prevention, 2018). While 72.6% of elderly in the UK got vaccinated, only 48.9% of at‐risk individuals under 65 years‐old got vaccinated that winter (Public Health England, 2018).

Although many public health interventions have been implemented to foster vaccination by encouraging acceptance and/or reducing vaccine hesitancy, there is still mixed evidence on which of these interventions have succeeded (Dubé et al., 2015; Korn et al., 2020; Milkman et al., 2011, 2021; Sadaf et al., 2013; World Health Organization, 2020). Alarmingly low vaccination rates further demonstrate the need for better understanding regarding what influences an individual's decision to get vaccinated.

Social norms, defined as individuals' perception about what most others do (Cialdini et al., 1990), are amenable to interventions and thus can be instrumental to foster vaccination. However, the role norms play on vaccination behavior ultimately depends on the interplay between *bandwagoning* and *free‐riding* effects, and the overall direction of their impact is unclear in the context of vaccination behaviors. On one hand, messages about others vaccinating in an individual's social environment may signal a social norm, thus *encouraging* individuals to get vaccinated. These messages can produce imitation or *bandwagoning* effects whereby individuals get vaccinated in response to others' vaccination. On the other hand, social norms messages may also signal a low individual risk of infection, thus *discouraging* vaccination and potentially promoting *free‐riding*. The theory of prevalence‐elastic demand for prevention suggests that as prevalence of an infectious disease decreases (a direct result of higher vaccination coverage), demand for prevention (such as vaccination) falls (Geoffard & Philipson, 1996, 1997; Hauck, 2018; Posner, 1993). At low prevalence levels (high vaccination coverage), perceived individual risk of infection is low, compelling unvaccinated individuals to free‐ride by not getting vaccinated (Ibuka et al., 2014; Vietri et al., 2012).

The complex interplay between these effects in driving individual behavior is likely shaped by the level of the population vaccination coverage conveyed through social norms messaging since population coverage rates convey not only norms but also risks of infection. Therefore, the bandwagoning effect might prevail at some population coverage rates leading individuals to get vaccinated, but individuals might free‐ride at higher coverage rates. This makes predicting the overall impact on vaccination intention of a social norms message about population vaccination coverage rates ambiguous. As shown in more detail in our literature review section, the evidence of which effect prevails is mixed.

Moreover, individual perceptions may also shape responses to social norms interventions. Due to imperfect information on the actual population vaccination coverage, perceived (vs. actual) coverage rate–that is, the vaccination coverage rate subjectively *perceived* by the individual–is a key mechanism that can potentially interact with public health messages (Gorman et al., 2012; Lehmann et al., 2014; Takayanagi et al., 2007). Additionally, each person may have an individually *perceived* risk of infection under which they decide not to get vaccinated. Once the population has achieved a specific vaccination threshold, the individual may choose to free‐ride on the coverage provided by others' vaccinations because they believe their personal risk of infection is sufficiently low to not vaccinate. This perceived risk threshold may differ across individuals, suggesting that even at the same actual population vaccination coverage rate, and therefore the same objective risk of infection, one individual may decide to get vaccinated while another may not.

Therefore, it is important to ascertain the role social norms messages play in vaccination decisions, and the heterogeneity of their effects in terms of the communicated population coverage rates, the perceived coverage rates, and the perceived risk of infection. To the best of our knowledge, no experimental study has yet systematically examined these aspects.

Therefore, in this paper we develop a theoretical model to study heterogeneity in vaccination behavior, and we empirically test the causal impact of messages about different population vaccination coverage rates on influenza vaccination intention. Using an online experiment with *N* = 1365 UK residents, we randomly allocate participants to receive a message of a vaccination coverage rate ranging between 10% and 95%, or to a control group with no such information. The impact of this treatment is measured via both self‐reported and behavioral measures of participants' influenza vaccination intention (Galizzi & Wiesen, 2018). Additionally, in order to gain insight into the heterogeneity of the treatment effects and the behavioral mechanisms underlying individual responses, we explicitly control for the respondents' perceived coverage rate, and the perceived risk of infection (i.e. the population vaccination rate at which individuals perceive the risk of infection to be sufficiently low to not get vaccinated). In traditional disease transmission epidemiological models often used when designing public health interventions, these behavioral factors are typically assumed constant and not explicitly modeled. They may, however, help explain how well‐intended social norms policies based on evidence from transmission models can fail to reach their target vaccination goals.

We find that groups treated with the information that the vaccination coverage rates in the population are at 65% or above have significantly higher average vaccination intention than the control group. For coverage rates up to 75%, individuals treated with higher coverage rates have greater vaccination intention than those treated with lower rates, consistent with a bandwagoning effect. However, for coverage rates above 75%, the treatment effects, albeit still positive, stop increasing and remain flat (or even decline), signaling that, besides a bandwagoning effect, a free‐riding effect may also kick in at high levels of the coverage rate. We also find significant heterogeneity of these effects depending on the individual perceptions of risks of infection and of the coverage rates.

The remainder of this paper is outlined as follows: Section 2 reviews the background literature, Section 3 presents a simple theoretical framework to model heterogeneity in vaccination decisions, Section 4 outlines the experimental design and the variables, Section 5 discusses the empirical methods, Section 6 presents the results of the experiment, and Section 7 concludes.

## 2 THE EXISTING LITERATURE AND CONTRIBUTION OF THIS STUDY

There is evidence that nudges based on social norms messages impact many health behaviors, including seatbelt and helmet wearing (Sunstein, 1996), risky sexual behavior (Sunstein, 1996), smoking, and obesity (Christakis & Fowler, 2007). These studies' social norms messages typically indicate that a specific proportion of the population adheres to the behavior (i.e. 80% of people wear their seatbelt while driving). A descriptive social norms message therefore sends a signal about the proportion of a population that engages in a certain behavior, which can help shape individual decisions by informing them of what is considered to be effective behavior (Cialdini et al., 1990). For example, Mahler et al. (2008) experimentally demonstrate that college students who are shown a descriptive norm message (that 85% of their peers use sunscreen regularly) have increased self‐reported sun protection behaviors compared to those not treated with the social norm. Similarly, Croker et al. (2009) demonstrate that male participants informed that 80% of British people attempt to eat at least five portions of fruit and vegetables per day have greater healthy eating intentions compared to men not exposed to this normative intervention.

With regards to vaccination uptake, evidence suggests that social norms messages correlate with vaccination behavior. Quinn et al. (2017) survey *N* = 1657 participants in the US to assess the relation between perceived social norm and self‐reported vaccination status: they find that perceived social norm is positively correlated with vaccination, suggesting *bandwagoning*. Chapman and Coups (1999) conduct two surveys (*N* = 79 employees at Rutgers University and *N* = 412 employees at a corporate workplace) to assess the relation between (self‐reported) norm and perceived risk of infection on self‐reported vaccination status. They find that perceived social norm is significantly predictive of vaccination, indicating a potential *bandwagoning* effect. Perceived risk of infection, however, is not significantly correlated with vaccination status. *Bandwagoning* effects are also found in the context of pregnant women (Gorman et al., 2012), healthcare workers (de Perio et al., 2012; Kraut et al., 2011; Takayanagi et al., 2007), and parents' decisions to vaccinate their children (Daley et al., 2007). *Bandwagoning* effects are also found in descriptive studies in other clinical areas, including human papillomavirus and hepatitis B vaccinations (Allen et al., 2009; Harmsen et al., 2012; Reiter et al., 2015). Some studies also assess the role of perceived risk of vaccination on uptake with mixed findings. Daley et al. (2007) find no effect, while a positive effect is found by Gorman et al. (2012). No study has systematically assessed the interplay between perceived risk of infection and social norms. Moreover, most studies are not designed to infer the causal impact of social norms on the uptake of vaccination.

From the few experimental studies assessing the causal impact of social norms on vaccination uptake, there is both evidence of a positive effect (*bandwagoning*) as well as a negative effect (*free‐riding*). Ibuka et al. (2014) experimentally model repeated social interactions between individuals where individuals learn their group's vaccination rate in the previous round. They find evidence in support of *free‐riding* behavior: individuals' likelihood to vaccinate decreases by 19 percentage points as the proportion of previous round vaccination among others increases from zero (none vaccinate) to one (all vaccinate). Böhm et al. (2016) investigate individual vaccination behavior in a dynamic and socially interactive game with full information about the incentive structure associated with the individual vaccination decision. While the authors find an average effect consistent with *free‐riding* (individuals' likelihood to vaccinate decreases as the proportion of others getting vaccinated increases), they also report that some individuals never free‐ride, some always free‐ride, and some conform to the norm or free‐ride depending on the incentive structure resulting from others' behavior. Hershey et al. (1994) also find evidence for both *bandwagoning* as well as *free‐riding* effects.

Additionally, individual perceptions of the social norm can play an important role in vaccination intentions and behavior, which can lead to heterogeneous responses to social norms interventions. Takayanagi et al. (2007) find that healthcare workers' belief that 50% or more of their colleagues vaccinate is positively associated with vaccine compliance. Böhm et al. (2016) find that 86% of participants in their experiment switch between vaccination and non‐vaccination depending on the incentives in each round of the experiment, but 26% never vaccinate regardless of the incentives and 7% always vaccinate. Heterogeneity in behavior related to bandwagoning and free‐riding is also found in more general contexts, including public good games (e.g. Fischbacher et al. (2001)).

While these studies do not fully unpack the factors associated with behavior heterogeneity, they do suggest that heterogeneity correlates with the composition of the group with which an individual interacts. Therefore, given that individuals' subjective perceptions of coverage rates play a role in decision‐making, they may also drive heterogeneity in response to coverage rates messages interventions even when the incentive structure is common knowledge.

How subjective perceptions of coverage rates relate to actual coverage rates can also impact decision‐making through a *surprise effect*. Previous studies on learning about HIV status establish that if an individual's perception does not match their reality, they are surprised by this difference and are prone to change their behavior (Baird et al., 2014; Boozer & Philipson, 2000; Gong, 2014). Individuals whose perceptions match their reality, however, are unsurprised and therefore are unlikely to change their behavior. Although HIV testing clearly differs from influenza vaccination, these findings suggest that vaccination intention depends on whether an individual's perception about the population coverage rate matches the intervention coverage rate. If they are equal or similar, the intervention is unlikely to have much impact on vaccination choices due to the lack of this surprise effect. If they differ, however, the intervention could have an effect on vaccination decisions.

The decision to vaccinate can also be affected by an individual's perceived risk of infection threshold, that is the population vaccination level at which they deem the risk of infection to be sufficiently low to stop demanding vaccination. Hershey et al. (1994) speculate that the bandwagoning effect they observe could be driven by the belief that the decision of others to vaccinate signals accurate information about risk of infection and therefore the need to vaccinate. Ibuka et al. (2014) report that individuals' perceived risk of infection decreases as they learn that others vaccinate, resulting in free‐riding. Brewer and Hallman (2006) interview a random sample of *N* = 300 Americans and find that perceived risk of infection is a more powerful predictor of self‐reported vaccination than objective risk of infection. They further report that perceived risk of infection fully mediates the impact of objective risk of infection on vaccination decisions. Chapman and Coups (1999) find that perceived risk of infection (measured through perceived likelihood of getting the flu) is positively correlated with likelihood of receiving the flu shot. In a meta‐analysis, Brewer et al. (2007) assess the role that perceived risks have in vaccination behavior by measuring perceived likelihood, susceptibility, and severity. While all perceived risks correlate positively with vaccination likelihood, they find the highest correlation with perceived risk of infection.

In summary, previous findings suggest that the effect of social norms on vaccination is ambiguous. Higher coverage rates conveyed in interventions correlate with the likelihood of vaccinating, but the overall effect depends on the extent to which the *bandwagoning effect* dominates the *free‐riding effect* at a given population coverage rate and on individual heterogeneity regarding perceptions. Given that perceptions play a key role in decision‐making, it is plausible that the way perceptions relate to coverage rates conveyed in interventions affect their ultimate impact on vaccination behavior. Previous studies are difficult to compare due to differences in study design, focus, and data. To the best of our knowledge, no study to date has systematically investigated the heterogeneous effect of different population vaccination coverage rates conveyed through social norms messaging. Notably, the literature assesses a limited range of coverage rates, and lacks consideration for the role that individual perceptions of population coverage rates and risk of infection play in decision‐making.

We therefore add to this literature in three significant ways: (i) we develop a theoretical framework to model heterogeneity on vaccination behavior; (ii) we assess the causal impact of social norms on self‐reported and behavioral vaccination intentions for a broad range of coverage rates messages; and (iii) we assess how the impact of messages interplays with individual perceptions about risk of infection and population coverage rates. Ultimately, the effectiveness of messages in promoting vaccination behavior may depend on the conveyed coverage rates, and the interplay between bandwagoning and free‐riding effects.

## 3 THEORETICAL FRAMEWORK

### 3.1 Perceived risk of infection (PRI)

We derive a simple theoretical framework that builds on the contribution by Betsch et al. (2013). Let $U_{V}$ and $U_{NV}$ denote the individual utility of vaccination (V) and not vaccination (*NV*), respectively, given by:

$$U_{V}\;\left(n\right)=B(H,\;C\left(n\right))-C_{V}$$

$$U_{NV}\;\left(n\right)=\gamma B\left(H_{D}\right)+\left(1-\gamma \right)B\left(H\right)$$

where n stands for the vaccination coverage rate, that is, the proportion of the population that get vaccinated, as informed by the experimental message; and γ stands for the perceived probability of getting infected ‐ that is the Perceived Risk of Infection, PRI ‐ when one is not covered by the vaccine, with γ=γ(n) and $\gamma_{n}^{\prime }\left(.\right)<0,\;\gamma_{n}^{\prime \prime }\left(.\right)>0$, that is, the perceived risk of infection decreases with the coverage rate at an increasing rate. The benefit B(h,C) denotes an additively separable function that captures the utility in terms of two arguments: a specific health status $h=\left\{H,H_{D}\right\}\;$ where $H_{D}$ stands for the average health status when infected by the flu and H for the health status when not infected, with $H_{D}\leq H$; the indirect benefit related to the number *n* of people vaccinating, that, for example, makes the flu less severe; and the psychological benefit of complying (C) with the social norm of vaccinating (SN), with $C_{n}^{\prime }\left(.\right)>0$. The additively separable function for the benefit function is assumed to satisfy standard properties $B^{\prime }\left(.\right)>0,\;B^{\prime \prime }\left(.\right)<0$. Finally, $C_{V}$ is the vaccine cost, that does not vary with *n* (Betsch et al., 2013).

An individual will vaccinate as long as $\Delta =U_{V}-U_{NV}\geq 0$ that is,

(1) $$\Delta =\gamma \left[B\left(H\right)-B\left(H_{D}\right)\right]+B(C\left(n\right))-C_{V}\geq 0$$

Assuming a random term ε in the individual's utility function that captures heterogeneous characteristics and unobservable factors affecting the vaccination uptake, Equation (1) can be written as

$$\Delta =\gamma \left[B\left(H\right)-B\left(H_{D}\right)\right]+B(C\left(n\right))-C_{V}+\varepsilon_{V}-\varepsilon_{NV}\geq 0$$

Assuming that the random terms $\varepsilon_{V},\;\varepsilon_{NV}$ are distributed as Extreme Value Type I (so that the difference between the vaccination and non‐vaccination decisions is logistically distributed), the probability of vaccinating is given by (see McFadden, 1974):

(2) $$\mathrm{Pr}\left(V\right)=\frac{e^{U_{V}}}{e^{U_{V}}+e^{U_{NV}}}=\frac{exp\left\{U_{V}-U_{NV}\right\}}{1+exp\left\{U_{V}-U_{NV}\right\}}$$

The probability of vaccinating is thus increasing with the perceived risk of infection, that is,

(3) $$\frac{\partial \mathrm{Pr}\left(V\right)}{\partial \gamma }=\frac{e^{\Delta }}{{\left(1+e^{\Delta }\right)}^{2}}\left[B\left(H\right)-B\left(H_{D}\right)\right]\geq 0$$

We can then assess the impact of the coverage rate n on the probability of vaccinating:

(4) $$\frac{\partial \mathrm{Pr}\left(V\right)}{\partial n}=\frac{e^{\Delta }}{{\left(1+e^{\Delta }\right)}^{2}}\frac{\partial \Delta }{\partial n}$$

The sign of Equation (4) depends on the sign of ∂Δ/∂n that is on how the difference in the utility of vaccinating minus non‐vaccinating changes with the coverage rate. With B(.) being additively separable and ∂γ/∂n<0 it simplifies to:

(5) $$\frac{\partial \Delta }{\partial n}=\frac{\partial B}{\partial C}\frac{\partial C}{\partial n}+\frac{\partial \gamma }{\partial n}\left[B\left(H\right)-B\left(H_{D}\right)\right]$$

where the first term is the marginal benefit of complying with the social norm (i.e. bandwagoning effect), whereas the second term is the opportunity cost of complying with the social norm (i.e. the cost of not free‐riding). Since $B^{\prime }\left(.\right)>0$, $C_{n}^{\prime }\left(n\right)>0$, and $\gamma_{n}^{\prime }\left(n\right)<0$ the sign of Equation (4) depends on the magnitude of these effects. Given the properties of these functions for low n the bandwagoning effect dominates the free‐riding effect, and therefore,

(6) $$\frac{\partial \mathrm{Pr}\left(V\right)}{\partial n}>0$$

that is the probability of vaccinating increases with the coverage rate. After a certain threshold of n, however, the free‐riding effect dominates the bandwagoning effect, thus implying

(7) $$\frac{\partial \mathrm{Pr}\left(V\right)}{\partial n}<0\;$$

that is the probability of vaccinating decreases with the coverage rate.

Finally, we can see how the impact of the coverage rate (i.e. the manipulated message about the coverage rate) on the probability of vaccinating varies with the perceived risk of infection:

(8) $$\frac{\partial^{2}\mathrm{Pr}\left(V\right)}{\partial n\partial \gamma }=\frac{e^{\Delta }}{{\left(1+e^{\Delta }\right)}^{3}}\left[\frac{\partial \Delta }{\partial n}\frac{\partial \Delta }{\partial \gamma }\frac{\left(1-e^{\Delta }\right)}{\left(1+e^{\Delta }\right)}\right]$$

With

$$\frac{\partial \Delta }{\partial \gamma }=\left[B\left(H\right)-B\left(H_{D}\right)\right]>0$$

Also $1-e^{\Delta }\leq 0$ for Δ≥0 (i.e., utility of vaccinating is greater than of not vaccinating). The sign of Equation (8) thus depends on ∂Δ/∂n. As shown above, for low n the bandwagoning effect dominates the free‐riding effect, and therefore, ∂Δ/∂n>0 while for high n ∂Δ/∂n<0. Therefore, for low n it follows that $\frac{\partial^{2}\mathrm{Pr}\left(V\right)}{\partial n\partial \gamma }<0$ while for high n $\frac{\partial^{2}\mathrm{Pr}\left(V\right)}{\partial n\partial \gamma }>0$ that is for low (high) manipulated coverage rates, the impact of the coverage rate on the probability of vaccinating decreases (increases) with the perceived risk of infection.

Therefore, the model posits the following research hypotheses:

*For* low levels of the coverage rates the probability of vaccinating increases with the coverage rate in the experimental message, while for high levels of the coverage rates the probability of vaccinating decreases with the coverage rate in the experimental treatment.

*The* probability of vaccinating increases with the perceived risk of infection.

*For* sufficiently low coverage rates (i.e. when the bandwagoning effect dominates the free‐riding effect) the effect of the coverage rate on the probability of vaccinating is higher for those with low perceived risk of infection than for those with high perceived risk of infection. For sufficiently high coverage rates (i.e. when the free‐riding effect dominates the bandwagoning effect) the effect of the coverage rate on the probability of vaccinating is higher for those with high perceived risk of infection than for those with low perceived risk of infection.

### 3.2 Perceived coverage rate (PCR)

Consider now that each individual has subjective beliefs about the coverage rates such that the Perceived Coverage Rate (PCR) $\hat{n}$ is a function of their beliefs β and of the coverage rate *n* manipulated by our experimental treatments, that is $\hat{n}\;=\;\frac{n}{\beta }$ with β>0. The term $\beta =\frac{n}{\hat{n}}$ therefore denotes both the direction and the magnitude of the perception of the coverage rate. The case β>1 denotes an individual who underestimated the number of people who vaccinate, that is, who thought the coverage rate was lower than the coverage rate in the experimental treatment (i.e. $\hat{n}<n$) with their underestimate increasing as β increases. On the other hand, the case β<1 denotes an individual who thought the coverage rate was higher than the coverage rate manipulated by the experimental message, thus overestimating the number of people who vaccinate (i.e. $\hat{n}\geq n\;$), with their ovestimate decreasing as β increases (i.e. as β→1). Finally the case β=1 denotes an individual who perceived the coverage rate exactly at the level of the coverage rate manipulated by the treatment. Then an individual will vaccinate as long as:

$$\Delta =\gamma \left[B\left(H\right)-B\left(H_{D}\right)\right]+B(C\left(\hat{n}\right))-C_{V}\geq 0$$

With $\gamma =\gamma \left(\hat{n}\right)$ and $\gamma_{\hat{n}}^{\prime }\left(.\right)<0,\;\gamma_{\hat{n}}^{\prime \prime }\left(.\right)>0$, i.e., the perceived risk of infection decreases with the perceived coverage rate at an increasing rate. It thus follows that,

(9) $$\frac{\partial \text{Pr}\left(V\right)}{\partial n}=\frac{e^{\Delta }}{{\left(1+e^{\Delta }\right)}^{2}}\left[\frac{\partial \Delta }{\partial \hat{n}}\frac{\partial \hat{n}}{\partial n}\right]$$

The sign of Equation (9) ultimately depends on the sign of Equation (5). As for the case above, for low levels of the coverage rate n the bandwagoning effect dominates the free‐riding effect and therefore Equation (9) is positive: the probability of vaccinating increases with the coverage rate. The opposite holds for high levels of the coverage rates in the experimental messages.

To assess how this effect Equation (9) varies with the perceived risk of infection:

(10) $$\frac{\partial^{2}\mathrm{Pr}\left(V\right)}{\partial n\partial \beta }=\frac{e^{\Delta }}{{\left(1+e^{\Delta }\right)}^{2}}\left[\frac{\left(1-e^{\Delta }\right)}{\left(1+e^{\Delta }\right)}\frac{\partial \hat{n}}{\partial \beta }\frac{\partial \hat{n}}{\partial n}\left({\left(\frac{\partial \Delta }{\partial \hat{n}}\right)}^{2}+\frac{\partial^{2}\Delta }{\partial {\hat{n}}^{2}}\right))+\frac{\partial \Delta }{\partial \hat{n}}\frac{\partial \hat{n}}{\partial n\partial \beta }\right]>0$$

For high levels of the coverage rate n the bandwagoning effect dominates the free‐riding effect and therefore Equation (10) is positive: that is for each treatment level the impact of the manipulated rate is higher for those respondents with β<1 (i.e. individuals that overestimate the coverage rate manipulated by the experimental message), than for those for which β>1 (i.e. individuals that underestimate the coverage rate). For low levels of the coverage rate n the sign of Equation (10) depends on the magnitude and sign of the various effects.

Finally, differentiating the probability of vaccinating with respect to the parameter for the perceived coverage rate gives:

$$\frac{\partial \mathrm{Pr}(V)}{\partial \beta }=\frac{e^{\Delta }}{{\left(1+e^{\Delta }\right)}^{2}}\frac{\partial \Delta }{\partial \hat{n}}\frac{\partial \hat{n}}{\partial \beta }$$

We know $\frac{\partial \hat{n}}{\partial \beta }<0,\;\frac{\partial \gamma }{\partial \hat{n}}<0$ and $\frac{\partial \Delta }{\partial \hat{n}}=\frac{\partial B}{\partial C}\frac{\partial C}{\partial \hat{n}}+\frac{\partial \gamma }{\partial \hat{n}}\left[B\left(H\right)-B\left(H_{D}\right)\right]$ that is positive for low levels of the perceived coverage rate $\hat{n}$ and negative for high levels. Therefore, it follows that, for $\hat{n}$ sufficiently low (i.e., for sufficiently high β) $\frac{\partial \mathrm{Pr}\left(V\right)}{\partial \beta }<0$, while for $\hat{n}$ sufficiently high (i.e., for sufficiently low β) $\frac{\partial \mathrm{Pr}\left(V\right)}{\partial \beta }>0$.

This implies that, for a given manipulated coverage rate, *ceteris paribus*, when the perceived coverage rate is lower than the coverage rate manipulated by the treatment (i.e. when β>1) participants are less likely to vaccinate the less they underestimate the coverage rate (i.e. as β→1). In other words, given an experimental message, the participants who initially mildly underestimated the coverage rate in the population are less likely to be ‘treated’ by the experimental information about the coverage rate ‐ and are therefore less likely to react to that information by choosing to vaccinate–than the participants who initially strongly underestimated the coverage rate. The participants react positively to the experimental message about coverage rate ‐ by vaccinating more ‐ when the message surprises them the most, showing that their initial beliefs strongly underestimated the proportion of people getting the vaccine. In the message they are told that the proportion of people actually vaccinating is far above the percentage they had in mind, and that therefore they significantly misread the social norms about vaccination. The intention to conform with the social norm to vaccinate here is motivated by the urge to conform to what most people do (i.e. ‘the wisdom of the crowd’), consistently with the bandwagoning effect. In contrast, the participants who learn from the message that their initial beliefs were not too far below the actual proportion of people vaccinating, are less likely to react to the message by getting the vaccine, because they feel less pressure to conform with what other people do.

Instead, for a given manipulated coverage rate, *ceteris paribus,* when the perceived coverage rate is higher than the coverage rate manipulated by the treatment (i.e. when β∈[0,1]) participants are more likely to vaccinate the more they overestimated the coverage rate. In other words, given an experimental message, the participants who initially strongly overestimated the coverage rate in the population are more likely to be ‘treated’ by the experimental information about the coverage rate ‐ and are therefore more likely to react to that information by choosing to vaccinate–than the participants who initially only mildly overestimated the coverage rate. Once again, the participants react positively to the experimental message about coverage rate–by vaccinating more–when the message surprises them the most, but this time it is because the message shows them that their initial beliefs strongly overestimated the proportion of people getting the vaccine. In the message they are told that the proportion of people actually vaccinating is far below the percentage they had in mind, and that therefore society is far away from reaching herd immunity by vaccination, hence the urge to get the vaccines themselves. Rather than by the bandwagoning effect, here the intention to conform with the social norm to vaccinate is motivated by the desire to protect themselves from the risks of getting infected by the flu, which reduces the incentives to free ride. In contrast, the participants who learn from the message that their initial beliefs were not too far above the actual proportion of people vaccinating, are less likely to react to the message by getting the vaccine, because they feel already protected by such a high coverage.

Therefore, we can derive the following additional hypothesis:

*For* a given manipulated coverage rate, those participants whose perceptions are such that they underestimated the coverage rate (i.e. β>1) are less likely to vaccinate the less they underestimated the coverage rate (i.e. as β→1). Instead, for a given manipulated coverage rate, those participants whose perceptions are such that they overestimated the coverage rate (i.e. β∈[0,1]) are more likely to vaccinate the more they overestimated the coverage rate (i.e. as β→0).

*For* high manipulated coverage rates the effect of the manipulated coverage rate is higher for those participants who overestimate the coverage rate than for those who underestimate it.

In what follows we test hypotheses [Link], [Link], [Link], [Link], [Link] making use of data from an experiment in England.

## 4 EXPERIMENTAL DESIGN AND VARIABLES

We conduct an online experiment to test the effects of manipulated messages about population vaccination coverage rates on individuals' influenza vaccination intentions. This study was conducted between May 3 and August 20, 2018 using Prolific (www.prolific.co), a crowdsourcing platform that recruits participants for online experiments. Individuals aged 18‐years‐old and over residing in the UK were eligible to participate. The sample is stratified by gender and the experiment has three sections: (i) baseline questionnaire, (ii) experimental treatments and (iii) final questionnaire to assess self‐reported vaccination intention and three behavioral proxies for vaccination.

### 4.1 Experimental treatments

In the experimental section, participants are randomly allocated to either a control group or one of seven treatment groups that receive information on different manipulated vaccination coverage rates in their social circle – 10%, 25%, 50%, 65%, 75%, 85%, or 95%. In the control condition, participants receive no message regarding coverage rate. In each of the seven treatment groups, individuals are primed with the following message: “*Imagine that X% (X out of 100) of the people you spend the most time with outside of your household (represented as the red persons above) normally get the flu vaccine*”, where *X* is one of the seven coverage rates, represented numerically and in natural frequencies with the number of vaccinated individuals in red out of 100 gray individuals (Appendix Figure SA). The pilot studies (*N* = 149 from first pilot and *N* = 302 for second pilot) revealed that a wide variation in manipulated coverage rates (10% to 95%) was needed to study the impact of a range of treatments on vaccination intention. We used the means and standard deviations of the outcome measures collected in the first two pilots to calculate the appropriate sample sizes for each group in the experiment, assuming two‐sample means tests between the control and each treatment group, alpha = 0.05 and power = 0.80. Using Stata and G*Power, these calculations suggested a sample size of 150 participants in each group.

### 4.2 Outcome measures

After participants are exposed to their treatment, their vaccination intention is assessed in four ways: one self‐reported and three behavioral proxies for vaccination intention (Table 1). As in previous studies (Li et al., 2016; Vietri et al., 2012), we measure self‐reported vaccination intention (*Vax*_*ik*_) – the likelihood that participant *i* subject to treatment *k* demands the flu vaccine–from 0 = *Not likely* to 100 = *Extremely likely* (Appendix Figure SB1). The three behavioral proxies for vaccination intention are collected as follows: first, we gauge the participant's interest in viewing an online map of nearby locations to get the flu vaccine, *Map*_*ik*_, equal to one if participant *i* follows a link to open an online map of pharmacies within their postcode that offer the flu vaccine, 0 otherwise (Evans & Rand, 2019; Rand, 2016) (Appendix Figure SB2). Second, for those participants who open the map, we record the duration of time (seconds) they spend looking at the map, *Maptime*_*ik*_ (Evans & Rand, 2019; Rand, 2016) (Appendix Figure SB3). Third, following previous studies (Fenerty et al., 2012; Vervloet et al., 2012; Dubé et al., 2015; E. B. Larson et al., 1982), we ask participants to download a calendar reminder to get the flu vaccine at the beginning of the next flu season (*Cal*_*ik*_), equal to one if participant *i* requests the reminder, 0 otherwise (Appendix Figure SB4).

**TABLE 1:** Description of variables

Variables	Description	Measure
Outcome measures		
Vax	Participant's self‐reported stated likelihood of getting the flu vaccine	0%–100%
Map	If the participant looked at the online map of available nearby pharmacies to get the flu vaccine	1 if Yes, 0 otherwise
Maptime	Participant's time spent looking at map (conditional on the participant looking at the map)	Seconds
Cal	If the participant downloaded the calendar reminder to get the flu vaccine at the start of the next flu season	1 if Yes, 0 otherwise
Controls		
Socio‐demographic characteristics		
Age	Participant's age	Years
Gender	Participant's gender	1 if female, 0 if male
White	If the participant's ethnic group is White (White British, White non‐British)	1 if White, 0 otherwise
Asian	If the participant's ethnic group is Asian (Indian, Pakistani, Bangladeshi, Chinese, other Asian background)	1 if Asian, 0 otherwise
Black	If the participant's ethnic group is Black (African, Caribbean, other Black background)	1 if Black, 0 otherwise
Other	If the participant's ethnic group is other (mixed or multiple ethnic groups, other ethnic groups)	1 if other, 0 otherwise
Education	Participant's highest educational or school qualification achieved	1 if low (none mentioned, completed primary school, completed secondary school), 2 if medium (post‐secondary vocational training, post‐secondary academic below‐degree level qualification), 3 if high (bachelors or equivalent first degree qualification, post‐graduate studies)
Employment	Participant's current employment situation	1 if employed or a student, 0 if unemployed
Vaccination history & other behavioral attitudes		
Past vax	If the participant got the flu vaccine in the previous flu season	1 if yes, 0 otherwise
Individual benefit	How much the participant agrees that getting the flu vaccine can protect themselves against the flu	0 (Strongly disagree)–100 (Strongly agree)
Prosocial benefit	How much the participant agrees that getting the flu vaccine can protect others in their environment who do not get vaccinated against the flu	0 (Strongly disagree)–100 (Strongly agree)
Spillovers	How much the participant agrees that others in their environment getting the flu vaccine can protect themselves and others who do not get vaccinated against the flu	0 (Strongly disagree)–100 (Strongly agree)
Social perceptions		
Perceived risk of infection (PRI)	What percentage of the participant's environment does the participant think need to get the flu vaccine to protect themselves and others who do not get vaccinated against the flu	0%–100%
Perceived coverage rate (PCR)	What percentage of the participant's environment does the participant think normally get the flu vaccine?	0%–100%
PRI higher	Absolute difference between treatment and perceived risk of infection if perceived risk of infection is higher than the treatment	0%–100%
PRI lower	Absolute difference between treatment and perceived risk of infection if perceived risk of infection is lower than the treatment	0%–100%
PCR higher	Absolute difference between treatment and perceived coverage rate if perceived coverage is higher than the treatment	0%–100%
PCR lower	Absolute difference between treatment and perceived coverage rate if perceived coverage rate is lower than the treatment	0%–100%

Abbreviations: PCR, perceived coverage rate; PRI, perceived risk of infection.

### 4.3 Perceptions

Previous studies demonstrate that higher vaccination coverage in an individual's social circle, and the associated lowered risk of infection, is a determinant of vaccination uptake (Betsch et al., 2013; Betsch et al., 2017). However, to the best of our knowledge, no study assesses the specific population coverage rate at which individuals perceive personal infection risk sufficiently low to not vaccinate (*Perceived Risk of Infection* (*PRI*)), and how this relates to vaccination intention. Therefore, to test hypotheses H2 and H3, we ask participants, using a similar framing as previous studies, “*What percentage of the people in your environment do you think need to get the flu vaccine to protect you and others who do not get vaccinated against the flu?*”*,* measured from 0% = *No one* to 100% = *Everyone*.

Similarly, while previous studies explore the association between perceived social norms and influenza vaccine compliance, they do not explore the range of potential perceived coverage rates and how they might impact vaccination. Gorman et al. (2012) use “most pregnant women” as the perceived social norm for flu vaccination in their survey. This only explores perceived coverage rates at which the majority of an individual's surrounding population vaccinates, but does not explore the range of perceived coverage rates, including those at which the minority vaccinate.

Therefore, to test hypotheses H4 and H5, following previous literature (Chapman & Coups, 1999; Quinn et al., 2017; Takayanagi et al., 2007), we elicit participants' subjective belief on population coverage rate (*Perceived Coverage Rate* (*PCR*)) by asking *“What percentage of the people in your environment do you think normally get the flu vaccine?”*, from 0% = *No one* to 100% = *Everyone*.

Eliciting both perceptions allows us to quantify the relationship between treatment effect and individual perceptions as stated in H2–H5.

Hypotheses H2–H5 imply there may be heterogeneity in the impact of our intervention on participants' intention to vaccinate. In our theoretical framework this heterogeneity depends on the relations between the treatment coverage rates and the individual perceptions about the risk of infection and the coverage rate. Therefore, in order to understand these potential differences, we construct the four variables described below.

#### 4.3.1 Difference between perceived risk of infection and manipulated coverage rate

**FIGURE 1:**
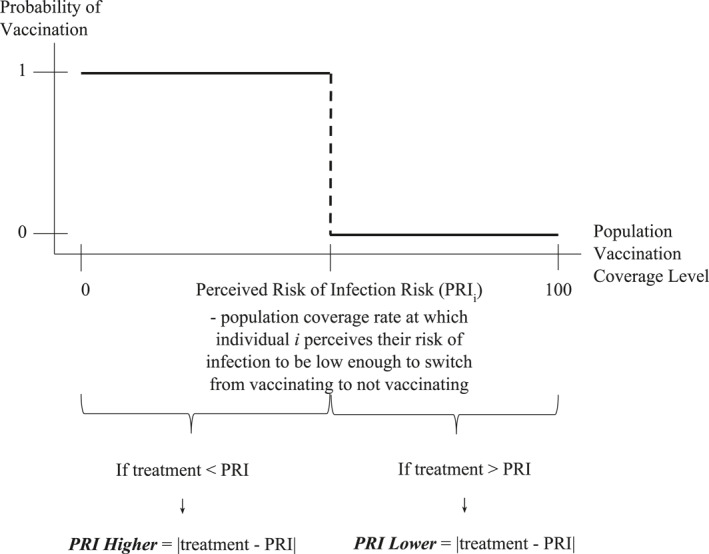
Description of perceived risk of infection variables: Perceived risk of infection (PRI) Higher and PRI Lower

Two factors that can affect vaccine uptake are i) whether the participant's perceived risk of infection is different than the manipulated coverage rate, and ii) if so, the distance between the participant's perceived risk of infection and the treatment rate. Therefore to empirically test H2 and H3, we construct two variables to account for the sign and the magnitude of the individual perceived risks of infection (Figure 1):

- ***PRI Higher*** *:* Equal to the absolute difference between the individual's perceived risk of infection and the manipulated coverage rate if the individual's perceived risk of infection is *higher* than their treatment coverage rate, 0 otherwise (i.e. if the perceived risk of infection is less than or equal to treatment coverage rate).
- ***PRI Lower*** *:* Equal to the absolute difference between the perceived risk of infection and the manipulated coverage rate if the perceived risk of infection is *lower* than treatment coverage rate, 0 otherwise (i.e. if the perceived risk of infection is greater than or equal to treatment coverage rate).

Those in the control group or whose perceived risk of infection is equal to their treatment are given a value of 0 for both *PRI* variables.

#### 4.3.2 Difference between perceived and manipulated coverage rates

Similarly, vaccination uptake can be affected by whether the participant's perceived coverage rate is different from the manipulated coverage rate and by the magnitude of that difference (if any). H4 posits that the effect of the perceived coverage rate depends on the direction and magnitude of the ratio between the manipulated and the perceived coverage rate. In particular, for a given manipulated coverage rate, those who underestimated the coverage rate (i.e. β>1) are less likely to vaccinate the less they underestimated the coverage rate (i.e. as β→1), whereas those who overestimated the coverage rate (i.e. β∈[0,1]) are more likely to vaccinate the more they overestimated the coverage rate (i.e. as β→0). H5 posits that for sufficiently high treatment levels the effect of the manipulated coverage rate is higher for those that overestimate the coverage rate.

**FIGURE 2:**
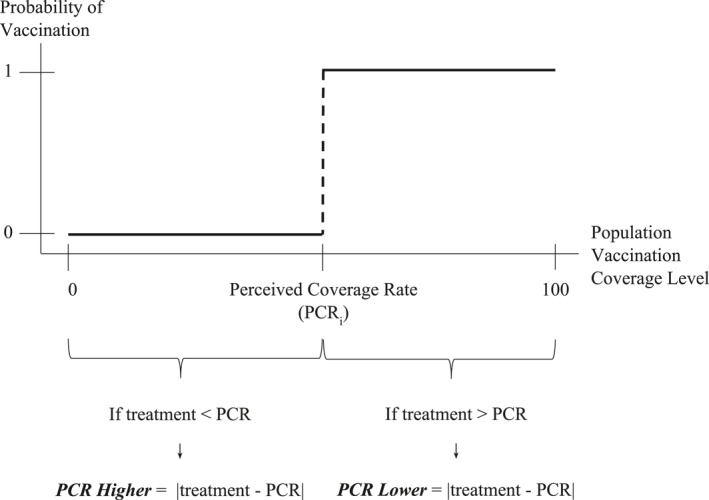
Description of perceived coverage rate variables: Perceived coverage rate (PCR) Higher and PCR Lower

Therefore to empirically test H4 and H5 we build two variables to account for the sign and the magnitude of the individual perceived coverage rates (Figure 2):

- ***PCR Higher:*** Equal to the absolute difference between the perceived coverage rate and the manipulated coverage rate if the perceived coverage rate is *higher* than treatment rate, 0 otherwise (i.e. if the perceived coverage rate is less than or equal to treatment coverage rate).
- ***PCR Lower*:** Equal to the absolute difference between the perceived coverage rate and the manipulated coverage rate if the perceived coverage rate is *lower* than treatment rate, 0 otherwise (i.e. if the perceived coverage rate is greater than or equal to treatment coverage rate).

Those in the control group or whose perceived coverage rate is equal to their treatment are assigned a value of 0 for both *PCR* variables.

Using these variables enables us to control not only for the underlying individual perceptions but also how these may interact with the treatment effect (heterogeneous treatment effects: see more below on empirical specifications). The latter is important because, as shown in Section 3, individuals may (partially or fully) update their beliefs about coverage rates after being exposed to the manipulated coverage rate. Importantly, the treatment's effect size may depend on the extent to which they are treated above or below their perceptions.

### 4.4 Controls

In the baseline questionnaire, we collect a variety of socio‐demographic characteristics, vaccination history, behavioral attitudes, and social perceptions correlated with vaccination (see Table 1 for all controls). Regarding socio‐demographic characteristics, age, gender, ethnicity, education, and employment have all been found to correlate with vaccination uptake (Brown et al., 2010; Chapman & Coups, 1999; Kohlhammer et al., 2007; H. J. Larson et al., 2014; Lu et al., 2015; Quinn et al., 2017; Schmid et al., 2017; Xakellis, 2005). *Age*_*i*_ is captured using participants' birth dates; *Gender*_*i*_ is measured as one if Female, 0 if Male; ethnicity is categorized as *White*_*i*_, *Asian*_*i*_, *Black*_*i*_, or *Other*_*i*_; *Education*_*i*_ is the participant's highest achieved educational qualification, grouped as low, medium, or high; *Employment*_*i*_ is valued at one if the participant is employed or a student, 0 if unemployed.

As previous studies (Chapman & Coups, 1999; Ward & Draper, 2008; Wheelock et al., 2014) find that past vaccinations correlate with future vaccination decisions, we ask participants about their vaccination status in the previous flu season (*Past Vax*_*i*_), equal to one if they vaccinated, 0 otherwise. Next, studies demonstrate that vaccination intentions can be affected by individual and prosocial benefits (Betsch et al., 2013, 2017; Li et al., 2016; Wheelock et al., 2017) as well as free‐riding attitudes (Hershey et al., 1994; Ibuka et al., 2014; Meszaros et al., 1996). All three factors are measured as the extent to which the subject agrees (from 0 = *Strongly Disagree* to 100 = *Strongly Agree*) with the following three statements. First, *Individual Benefit*_*i*_ is assessed using the statement, *“If you get the flu vaccine, you can protect yourself against the flu.”* Second, *Prosocial Benefit*_*i*_ is measured with the statement, *“If you get the flu vaccine, you can protect others in your environment who do not get vaccinated against the flu.”* Third, free‐riding (*Spillovers*_*i*_) is tested with the statement, *“If others in your environment get the flu vaccine, they can protect you and others who do not get vaccinated against the flu.”*

## 5 EMPIRICAL METHODS

We first estimate the average treatment effect for each vaccination intention outcome measure, using linear regression for stated vaccination intention, logit regression for map viewing interest, Cragg's double hurdle model (Cragg, 1971) for time spent looking at the map, and logit regression for calendar reminder download. Models are first run without covariates since this randomized experiment is designed to be well‐balanced in observed characteristics between treated and control groups. We additionally estimate these same models controlling for potential imbalances in observable covariates to reduce the residual variability in our outcome variables and thus obtain more precise estimates of the treatment effect.

### 5.1 Testing the hypotheses in the empirical models

To empirically test H1, we directly look at the estimated coefficients of the different coverage rates treatments. For hypothesis H1 to be supported by our empirical model, we expect to see the estimated coefficients for the different treatments increasing up to a certain level of the coverage rate, and then stopping increasing and remaining flat, or even decreasing, thereafter.

To empirically test H2, we look at the estimated coefficients of the above described variables *PRI Higher* and *PRI Lower* when they are included in the above regressions as control variables. For hypothesis H2 to be supported by our empirical model, we expect to see the estimated coefficients of *PRI Higher* and *PRI Lower* being positive and negative, respectively, and statistically significant.

To empirically test H3, we look at the estimated coefficients of the different coverage rates treatments in two sets of regressions where we stratify the participants into two groups according to whether they have low or high perceived risks of infection as measured by the variables *PRI Higher* and *PRI Lower*, respectively. For hypothesis H3 to be supported by our empirical model, we expect to see the estimated coefficient for each treatment for the *PRI Lower* respondents being higher than the corresponding coefficient for the *PRI Higher* participants when the coverage rates are low, and the reverse when the coverage rates are high.

To empirically test H4 and H5, we look at the estimated coefficients of the variables *PCR Lower* and *PCR Higher* in two sets of regressions where we stratify the participants into two groups according to whether they have low or high perceived coverage rates as measured by those variables, and where we control for the different coverage rates treatments. For hypothesis H4 to be supported by our empirical model, we expect to see the estimated coefficient for *PCR Lower* being negative and statistically significant, and the estimated coefficient for *PCR Higher* being positive and statistically significant. For H5 to be supported we expect the estimated coefficient of the manipulated coverage rates to be larger for those with high PCR (i.e. those for which *PCR Higher* > 0) than for those with low PCR (i.e. those for which *PCR Lower* > 0).

### 5.2 Stated vaccination intention

The impact of the manipulated coverage rates on stated flu vaccination intention ($Vax_{i}$) for participant *i* is modeled as:

(11) $$Vax_{ik}=\;\alpha +\;\beta_{k}T_{ik}+\gamma P_{i}\;+\delta X_{i}+\varepsilon_{i}$$

where α is the constant, *T*_*ik*_ is a vector of our seven treatment variables taking the value of one if participant *i* is treated with the specified social norm *k*, 0 otherwise. Each treatment group is compared against the control group for which *T*_*ik*_ = 0, ∀i. Vector *P*_*i*_ contains *PRI Higher, PRI Lower, PCR Higher,* and *PCR Lower*. Vector *X*_*i*_ contains socio‐demographic (*Age, Gender, Ethnicity, Education, Employment*), vaccination history (*Past Vax*), and behavioral attitudes (*Individual Benefit, Prosocial Benefit, Spillovers*) controls; $\varepsilon_{i}$ is the error term.

### 5.3 Map interest

We specify a logit model to measure the impact of social norms messaging on map interest ($Map_{ik}$) as:

(12) $$\text{logit}\left(\pi_{ik}\right)=\log\left(\frac{\pi_{ik}}{1-\pi_{ik}}\right)=\alpha +\beta_{k}T_{ik}+\gamma P_{i}\;+\delta X_{i}+\varepsilon_{i}$$

where $\pi_{ik}=\text{Pr}\left({\text{Map}}_{ik}=1|T_{ik},P_{i},\;X_{i}\right)$ (see Appendix C1 in Supporting Information for log‐likelihood function). The other variables and parameters are defined as above.

### 5.4 Time expended looking at the map

Since the outcome variable that measures the length of time in seconds respondent *i* in treatment group *k* spends looking at the map (*Maptime*_*ik*_) is only observed for those who choose to view the map in the first place, this creates two issues. First, relatively few participants chose to view the map (*N* = 325), leaving *N* = 1040 zero‐value observations for time spent on the map, leading to potential censoring. Second, as the time spent looking at the map is predicated on the decision to look at the map, it cannot be modeled in one‐stage as this would confound the first stage decision. Thus, using traditional linear regression for *Maptime*_*i*_ would lead to underestimated β coefficients. To overcome these issues, we use a Cragg's double hurdle model (Cragg, 1971) as it allows the explanatory factors affecting the participation decision to differ from those affecting the time expended decision (i.e. extensive vs. intensive margins). The model is specified following the literature (Blundell & Meghir, 1987; Eakins, 2016; Newman et al., 2003), as:

(13) $${y^{*}}_{1ik}=\omega D_{ik}+\varepsilon_{i}\;\text{Participation}\;\text{Decision}$$

(14) $${y^{*}}_{2ik}=\omega D_{ik}+{𝑢}_{i}\;\text{Time}\;\text{Expended}\;\text{Decision}$$

(15) $$Maptime_{ik}=\;{y^{*}}_{2ik}\;\text{if}\;{y^{*}}_{1ik}=1\;\text{and}\;{y^{*}}_{2ik}>0$$

(16) $$Maptime_{ik}=0\;\text{otherwise}$$

where $\omega D_{ik}=\beta_{k}T_{ik}+\gamma P_{i}\;+\delta X_{i}$, ${y^{*}}_{1ik}$ is a latent variable characterizing the participation decision (look at the map or not), ${y^{*}}_{2ik}$ is a latent variable characterizing the time expended decision. $Maptime_{ik}$ is the observed dependent variable (time expended looking at the map), equal to ${y^{*}}_{2ik}$ if both hurdles are passed (i.e. ${y^{*}}_{1ik}=1$ and ${y^{*}}_{2ik}>0$) and 0 otherwise; error terms, $\varepsilon_{i}$ and $u_{i}$, are independent and normally distributed. See Appendix C2 in Supporting Information for log‐likelihood function.

### 5.5 Calendar reminder

Finally, participant *i* in treatment group *k*'s decision to download a calendar reminder to get the flu vaccine $\left(Cal_{ik}\right)$, is modeled via a logit model specified as:

(17) $$\text{logit}\left(\rho_{ik}\right)=\log\left(\frac{\rho_{ik}}{1-\rho_{ik}}\right)=\alpha +\beta_{k}T_{ik}+\gamma P_{i}\;+\delta X_{i}+\varepsilon_{i}$$

where $\rho_{ik}=\text{Pr}\left({\text{Cal}}_{ik}=1|T_{ik},P_{i},\;X_{i}\right)$ (see Appendix C3 in Supporting Information for log‐likelihood function).

### 5.6 Overview of the empirical models

Ten empirical models are run for each vaccination intention measure. Model 1 (M1) is the average treatment effect, followed by five regression models: model 2 (M2) includes dummies for all treatment groups; model 3 (M3) is M2 with socio‐demographic controls; model 4 (M4) is M3 with vaccination history and behavioral attitudes controls. We further run empirical model 5 (M5), that is the same as M4 but also includes *Perceived Risk of Infection* and *Perceived Coverage Rate* as control variables. Empirical models M1‐M5 test hypothesis H1.

To test H2 we run empirical model 6 (M6), that is the same as M4 but also includes *PRI Higher* and *PRI Lower* as control variables.

To test H3 we run empirical models 7 and 8 (M7, M8) which stratify the participants into two groups with high and low perceived risk of infection as measured by *Perceived Risk of Infection*, respectively, and we compare the estimates of the coefficients of the coverage rates treatments across M7 and M8.

To test H4 we run empirical models 9 and 10 (M9, M10) which stratify the participants into two groups with high and low perceived coverage rates as measured by *Perceived Coverage Rate*, respectively, and we look at the estimated coefficients of the control variables *PCR Higher* and *PCR Lower* in M9 and M10 respectively.^1^ To test H5 we compare the treatment effects between M9 and M10.

## 6 RESULTS

### 6.1 Descriptive statistics

All *N* = 1365 participants are used to analyze models 1–3 for each vaccination intention measure. Since one pilot study (*N* = 149) does not include data on behavioral attitudes and perceptions, models 4–6 use only *N* = 1216 participants. Models 7‐10 stratify participants based on *Perceived Risk of Infection* and *Perceived Coverage* Rate and thus include *N* = 813, 556, 483, and 886 participants, respectively. Participants are stratified by gender: 683 (50%) male and 682 (50%) female. The mean (M) duration of experiment participation is 8.5 min (standard deviation [SD] = 2.7). The mean age of all participants is 36.7 years (SD = 12). Most participants are White British (*N* = 1230%; 90%), followed by Asian (*N* = 70; 5.1%), Black (*N* = 24; 1.8%) and Other (*N* = 41, 3%), aligning with UK Census findings (Office for National Statistics; National Records of Scotland; Northern Ireland Statistics and Research Agency, 2016). Subjects' education levels are split between low (*N* = 296; 21.7%), medium (*N* = 314; 23%), and high (*N* = 755; 55.3%).

**FIGURE 3:**
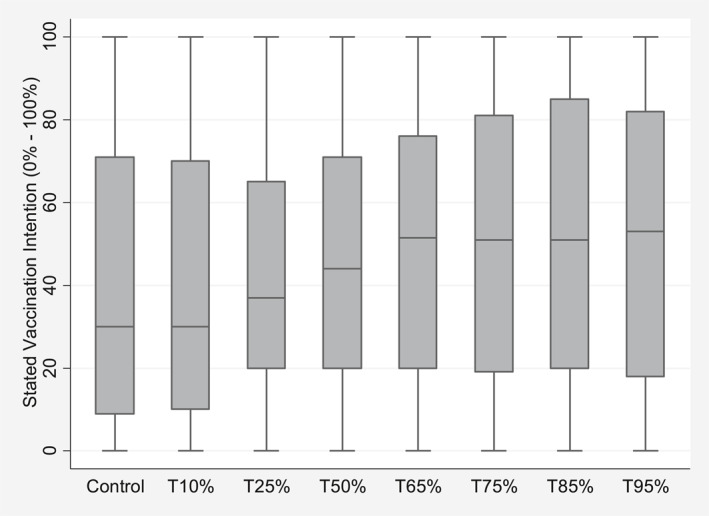
Boxplot for *Vax*_*ik*_, participant *i*'s stated vaccination intention by control and treatment groups *k*, unadjusted for covariates. Different treatment groups state the percentage of others in the participant's environment that normally get the flu vaccine (i.e. T10% refers to 10%, T25 refers to 25%, etc.)

**FIGURE 4:**
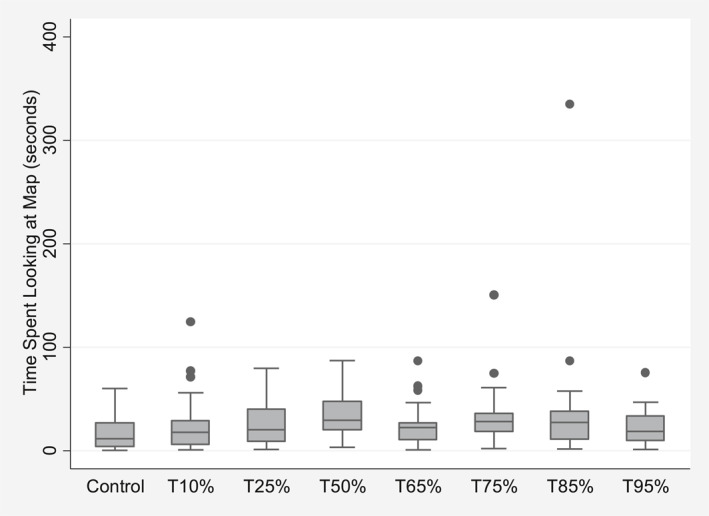
Boxplot for *Maptime*_*ik*_, the time participant *i* spends looking at the map given their interest in the map by control and treatment groups *k*, unadjusted for covariates. Different treatment groups state the percentage of others in the participant's environment that normally get the flu vaccine (i.e. T10 refers to 10%, T25 refers to 25%, etc.)

The mean self‐reported vaccination intention (*Vax*_*ik*_) across all participants is 46.5% (SD = 34.2) (see Figure 3 for distributions by treatment group). The 325 (23.81%) participants who observe the map (*Map*_*ik*_) spend on average 26.8 s (SD = 26.6) looking at it (*Maptime*_*ik*_) (see Figure 4 for distributions by treatment group). There are 133 (9.7%) participants who downloaded the calendar reminder (*Cal*_*ik*_). Across all participants, the average perceived risk of infection is 64.46% (SD = 30.15). Respondents' average perceived risk of infection is either 17.5% (SD = 25.1) greater than or 11.2% (SD = 21) lower than their treatment coverage rate, depending on if their perceived risk of infection lies above (*PRI Higher*_*i*_) or below (*PRI Lower*_*i*_) their treatment. The average perceived coverage rate is 37.72% (SD = 22.84) across all groups. Respondents' average perceived coverage rate is either 6.4% (SD = 14.4) greater than or 24.2% (SD = 26.5) lower than their treatment coverage rate, depending on if their perceived coverage rate lies above (*PCR Higher*_*i*_) or below (*PCR Lower*_*i*_) their treatment. See Table 2 for all descriptive statistics.

**TABLE 2:** Descriptive statistics

Variables	Total no. of participants	Mean/No. (%) of participants	SD	Min	Max
Outcome measures					
Vax	1365	46.50	34.22	0	100
Map (No. and % interested)	1365	325 (23.81%)			
Maptime	325	26.78	26.60	0.57	334.88
Cal (No. and % downloaded)	1365	133 (9.74%)			
Controls					
Socio‐demographic characteristics					
Age	1365	36.73	12.03	18	77.53
Gender					
Female	1365	682 (49.96%)			
Male	1365	683 (50.04%)			
Ethnicity					
White	1365	1230 (90.11%)			
Asian	1365	70 (5.13%)			
Black	1365	24 (1.76%)			
Other	1365	41 (3%)			
Education					
Low	1365	296 (21.68%)			
Medium	1365	314 (23%)			
High	1365	755 (55.31%)			
Employment					
Employed	1365	1241 (90.92%)			
Unemployed	1365	124 (9.08%)			
Vaccination history & behavioral attitudes					
Past vax (% vaccinated)	1365	299 (21.90%)			
Individual benefit	1216	70.56	25.71	0	100
Prosocial benefit	1216	61.31	29.98	0	100
Spillovers	1216	59.33	29.38	0	100
Perceptions					
Perceived risk of infection	1216	64.46	30.15	0	100
Perceived coverage rate	1216	37.72	22.84	0	100
PRI higher	1216	17.54	25.09	0	90
PRI lower	1216	11.16	20.99	0	95
PCR higher	1216	6.41	14.43	0	90
PCR lower	1216	24.20	26.49	0	95

*Note*: Behavioral attitude controls and social perception data was not collected from participants in the first pilot study (*N* = 149); thus, reducing the total number of participants from 1365 to 1216 for those variables (Individual Benefit, Prosocial Benefit, Spillovers, Perceived Risk of Infection, Perceived Coverage Rate, PRI Higher, PRI Lower, PCR Higher, and PCR Lower).

Abbreviations: PCR, perceived coverage rate; PRI, perceived risk of infection.

Kruskal‐Wallis (Appendix Table SD) and Mann‐Whitney tests (Appendix Table SE) show no statistical differences between the control group and individual treatment groups for most socio‐demographic variables, as expected in a randomized experiment. We only find slight imbalances for age between the control group and the following treatment groups: 25% coverage level (M_B‐A_ = 3; *Z* = −3, *p* < 0.01), 50% coverage level (M_B‐A_ = 1.7; *Z* = −1.8, *p* = 0.07), 75% coverage level (M_B‐A_ = 2.5; *Z* = −2.4, *p* = 0.01), 85% coverage level (M_B‐A_ = 3.8; *Z* = −3.3, *p* < 0.01), 95% coverage level (M_B‐A_ = 2; *Z* = −1.9, *p* = 0.06). We thus control for age in our regressions.

### 6.2 Vaccination intentions

We find that treating individuals with vaccination coverage rates has statistically significant positive effects on vaccination intentions. The average effects in our samples vary across measures and tend to be positive and statistically significant only at higher manipulated rates, particularly once controls are added.

#### 6.2.1 Self‐reported intention

Results for all models (Table 3, M1‐M4) show that a manipulated coverage rate of 65% and above has a significant positive impact on self‐reported vaccination intention. We find evidence in support of hypothesis H1: the effects of the manipulated coverage rates are positive (albeit not always significant) and increase up to a 75% coverage rate. However, for coverage rates above 75% the treatment effects, albeit still positive, stop increasing and remain flat (or even decline). This suggests that, as coverage rates increase, besides a bandwagoning effect, a free‐riding effect may also kick in.

**TABLE 3:** OLS estimates of the impact of social norms messaging on stated vaccination likelihood (Vax)

Variables	M1 ATE	M2 TG	M3 SDC	M4 VBC	M5 PER	M6 DIST	M7 High PRI	M8 Low PRI	M9 High PCR	M10 Low PCR
Social norm message treatment										
10%	0.576	1.092	1.459	0.139	0.292	−7.899*	0.420	−2.927	−3.510	−7.056
	(3.425)	(3.644)	(3.651)	(3.107)	(3.058)	(4.437)	(3.134)	(9.429)	(3.861)	(8.970)
25%	1.785	1.101	1.225	1.700	2.024	−3.029	3.325	−6.444	−1.885	4.718
	(3.611)	(3.375)	(3.377)	(3.102)	(3.056)	(3.801)	(3.203)	(6.660)	(3.718)	(4.422)
50%	3.030	3.030	3.368	3.402	3.517	4.138	3.496	4.422	4.496	5.244
	(3.670)	(3.664)	(3.666)	(3.111)	(3.062)	(3.381)	(3.371)	(4.832)	(4.385)	(3.824)
65%	10.18***	7.210**	7.325**	9.070***	9.142***	13.19***	11.28***	6.452	13.66**	13.55***
	(3.347)	(3.664)	(3.670)	(3.116)	(3.073)	(3.509)	(3.572)	(4.168)	(6.206)	(3.853)
75%	9.797***	12.11***	12.51***	9.577***	9.555***	16.08***	15.34***	4.322	−2.265	16.09***
	(3.787)	(3.387)	(3.391)	(3.115)	(3.066)	(3.659)	(3.807)	(3.833)	(8.886)	(3.939)
85%	10.07***	10.07***	10.64***	9.876***	9.433***	18.28***	10.43***	10.40***	21.63	16.96***
	(3.794)	(3.651)	(3.662)	(3.113)	(3.072)	(3.912)	(4.004)	(3.631)	(15.26)	(4.219)
95%	9.098**	9.098**	9.731***	9.597***	9.047***	20.55***	16.09***	8.158**	21.60*	17.68***
	(3.797)	(3.644)	(3.654)	(3.106)	(3.060)	(4.205)	(4.952)	(3.337)	(12.99)	(4.532)
Socio‐demographic controls										
Age			−0.100	−0.150**	−0.135**	−0.145**	−0.173**	−0.140	−0.0831	−0.178**
			(0.0803)	(0.0695)	(0.0685)	(0.0690)	(0.0851)	(0.0999)	(0.102)	(0.0830)
Gender			2.686	3.080*	2.069	2.291	2.358	3.727	1.845	3.029
			(1.848)	(1.581)	(1.565)	(1.574)	(1.930)	(2.329)	(2.374)	(1.883)
Asian			8.518**	6.312*	5.331	6.061*	5.617	11.46**	12.25**	3.543
			(4.221)	(3.578)	(3.524)	(3.544)	(4.096)	(5.576)	(5.004)	(4.346)
Black			0.766	1.174	−0.512	−0.157	6.839	−8.540	−5.520	2.375
			(7.049)	(5.747)	(5.668)	(5.703)	(7.292)	(8.693)	(8.711)	(7.248)
Other			−4.672	−0.499	0.287	−0.471	1.079	−2.233	6.829	−2.633
			(5.419)	(4.556)	(4.485)	(4.514)	(5.599)	(6.707)	(7.207)	(5.321)
Education			0.694	−1.358	−1.097	−1.159	−2.222*	−0.893	−1.654	−0.946
			(1.150)	(0.983)	(0.978)	(0.981)	(1.202)	(1.432)	(1.453)	(1.181)
Employment			−1.278	1.495	1.520	1.406	0.227	2.619	3.491	0.541
			(3.334)	(2.765)	(2.721)	(2.736)	(3.464)	(3.802)	(4.201)	(3.248)
Vaccination history & behavioral attitudes controls										
Past vax				39.42***	37.20***	37.77***	41.09***	41.58***	43.74***	39.22***
				(1.934)	(1.935)	(1.953)	(2.264)	(3.028)	(2.865)	(2.347)
Individual benefit				0.330***	0.316***	0.317***	0.295***	0.323***	0.251***	0.378***
				(0.0395)	(0.0398)	(0.0398)	(0.0518)	(0.0524)	(0.0598)	(0.0461)
Prosocial benefit				0.0182	−0.00368	−0.00646	0.0313	−0.0247	−0.00889	0.0239
				(0.0491)	(0.0485)	(0.0489)	(0.0578)	(0.0794)	(0.0755)	(0.0588)
Spillovers				0.0661	0.0414	0.0517	0.0416	0.0931	0.108	0.0319
				(0.0470)	(0.0465)	(0.0467)	(0.0541)	(0.0794)	(0.0693)	(0.0573)
Perception controls										
Perceived risk of infection					0.120***					
					(0.0290)					
Perceived coverage rate					0.155***					
					(0.0348)					
PRI higher						0.0978**				
						(0.0495)				
PRI lower						−0.143***				
						(0.0459)				
PCR higher						0.111			0.150*	
						(0.0734)			(0.0813)	
PCR lower						−0.120**				−0.150***
						(0.0487)				(0.0567)
Constant		41.16***	42.43***	9.345*	−0.463	12.69**	16.24**	7.255	9.508	8.880
		(2.392)	(5.856)	(5.590)	(5.780)	(5.653)	(7.072)	(7.390)	(7.948)	(6.481)
Observations	1365	1365	1365	1216	1216	1216	813	556	483	886
R‐squared		0.018	0.024	0.390	0.411	0.405	0.413	0.403	0.461	0.399

*Note*: Standard errors in parentheses; ***p < 0.01, **p < 0.05, *p < 0.1; Participants from the first pilot study (*N* = 149) are excluded from models 4‐6 as data on their behavioral attitudes and social perceptions were no tcollected, leaving the remaining 1216 participants for analysis; Models are as follows–M1: average treatment effect (ATE), M2: treatment group dummies (TG), M3: M2 + socio‐demographic controls (SDC), M4: M3 + vaccination history & behavioral attitudes controls (VBC), M5: M4 + Perceived Risk of Infection + Perceived Coverage Rate (PER), M6: M4 + PRI Higher/Lower + PCR Higher/Lower (DIST), M7: M4 stratified by high PRI, M8: M4 stratified by low PRI, M9: M4 stratified by high PCR, M10: M4 stratified by low PCR.

Abbreviations: PCR, perceived coverage rate; PRI, perceived risk of infection.

These effects are robust to the introduction of control variables accounting for the individual perceptions of the risk of infection and of the coverage rate (M5). Both underlying perception variables (i.e. *Perceived Risk of Infection* and *Perceived Coverage Rate*) have positive and significant coefficients, suggesting higher stated likelihood of vaccination with greater perceived risk or perceived coverage rate (M5).

Once we add the difference between perceptions and treatment coverage rates (*PRI Higher/Lower, PCR Higher*/*Lower*) in M6, the effect on self‐reported vaccination intention is markedly greater compared to M5 (Table 3). Effects are greater at coverage rates of 65% and higher, and are particularly large for those treated with the two highest rates (85% and 95%). They have 18.3% (*p* < 0.01) and 20.6% (*p* < 0.01) (M6) higher vaccination intentions than the control group, respectively. *T*‐tests comparing treatment groups incrementally (i.e. 10% vs. 25%, 25% vs. 50%, etc.) show significant differences in self‐reported vaccination intention only between the 50% (*M* = 44.2, SD = 2.7) and the 65% (*M* = 51.4, SD = 2.2) groups (t(348) = −2.1, *p* = 0.04). The group treated with the lowest manipulated coverage rate (10%) has 7.9% (*p* = 0.07) lower stated vaccination intention compared to the control group, suggesting that such a low coverage rate may induce a behavior change in the opposite direction (i.e. weakened vaccination intention); however, this finding is only marginally significant.

The positive coefficient of 0.10 (*p* = 0.05) for *PRI Higher* indicates that respondents whose perceived risk of infection is greater than their treatment have higher self‐reported vaccination intention compared to individuals in the control group or whose perceived risk of infection is equal to their treatment, and who therefore are effectively untreated (M6). Furthermore, as the difference between perceived risk of infection and treatment increases (i.e. *PRI Higher* becomes larger), individuals' likelihood to vaccinate also increases. The negative coefficient of −0.14 (*p* < 0.01) for *PRI Lower* correspondingly suggests that respondents whose perceived risk of infection is less than the treatment have lower self‐reported vaccination intention compared to respondents in the control group or whose perceived risk of infection is equal to their treatment. This provides evidence in support of hypothesis H2.

For perceptions of coverage rates (M6), the negative coefficient of −0.12 (*p* = 0.01) for *PCR Lower* indicates that participants whose perceived coverage rate is less than their treatment have lower self‐reported vaccination intention compared to participants in the control group or whose perceived coverage rate is equal to the treatment. The effect of *PCR Higher* is not statistically significant.

As it can be seen by comparing the estimates in M7 and M8, with the only exception of the 50% coverage rate, the estimated coefficients of the coverage rates treatments are always higher for the group of participants with higher perceived risk of infection (M7) than the corresponding estimates for the participants with lower perceived risk of infection (M8). This provides limited evidence in support of hypothesis H3: as explained above, in fact, H3 postulates that the coefficients for the coverage rate treatments are higher for the participants with higher perceived risk of infection than for the respondents with lower risk of infection only when the coverage rates are sufficiently high (which holds here), while it postulates that the reverse is true when the coverage rates are low (which is not the case here). In any case, this set of stratified estimations allows us to highlight the significant heterogeneity of the treatment effects when these are interacted with the different perceptions of the risk of infection.

When we stratify the participants into two groups with high and low perceived coverage rates in M9 and M10, respectively, the estimated coefficients of the control variables *PCR Higher* and *PCR Lower* are positive and negative, respectively, and statistically significant. This provides some evidence in support of hypothesis H4, although it should be noted that the estimated coefficient for the *PCR Higher* is only marginally significant. Finally with regards to H5 we can see that the estimated coefficients of the coverage rates, when significant, tend to be larger for those with a high PCR than for those with a low PCR for sufficiently high manipulated coverage rates. Also this other set of stratified estimations allows us to highlight the significant heterogeneity of the treatment effects when these are interacted with the different perceptions of the coverage rates.

#### 6.2.2 Behavioral measures for vaccination intention

In models 1–5 (Table 4), every treatment vaccination coverage rate has a statistically significant positive impact on the interest in looking at the map of available flu vaccine locations when compared to the control group. Also for the interest in looking at the map we find evidence in support of hypothesis H1: the effects of the manipulated coverage rates is positive (albeit not always significant) and increase up to a 75% coverage rate. However, for coverage rates above 75% the treatment effects, albeit still positive, stop increasing and remain flat (or even decline), suggesting that, as coverage rates increase, besides a bandwagoning effect, a free‐riding effect may also kick in.

**TABLE 4:** Average marginal effects from a logit regression of the impact of social norms messaging on interest in looking at the map of influenza vaccination locations (Map)

Variables	M1	M2	M3	M4	M5	M6	M7	M8	M9	M10
	ATE	TG	SDC	VBC	PER	DIST	High PRI	Low PRI	High PCR	Low PCR
Social norm message treatment										
10%	0.0967**	0.116**	0.108**	0.128**	0.131**	−0.00990	0.133**	0.129	0.107*	−0.0167
	(0.0420)	(0.0498)	(0.0488)	(0.0528)	(0.0526)	(0.0757)	(0.0553)	(0.153)	(0.0573)	(0.177)
25%	0.111***	0.130***	0.122***	0.141***	0.141***	0.0397	0.146***	0.136	0.0959*	0.156**
	(0.0389)	(0.0463)	(0.0454)	(0.0520)	(0.0518)	(0.0653)	(0.0549)	(0.0966)	(0.0556)	(0.0655)
50%	0.0814*	0.100**	0.0962*	0.116**	0.119**	0.0720	0.158***	−0.0863	0.160**	0.0805
	(0.0417)	(0.0506)	(0.0495)	(0.0535)	(0.0533)	(0.0583)	(0.0572)	(0.104)	(0.0625)	(0.0630)
65%	0.135***	0.152***	0.139***	0.160***	0.159***	0.142**	0.175***	0.116**	−0.0705	0.181***
	(0.0438)	(0.0487)	(0.0478)	(0.0518)	(0.0517)	(0.0573)	(0.0586)	(0.0563)	(0.120)	(0.0590)
75%	0.150***	0.166***	0.167***	0.213***	0.215***	0.216***	0.248***	0.159***	0.256**	0.217***
	(0.0402)	(0.0454)	(0.0444)	(0.0503)	(0.0501)	(0.0576)	(0.0589)	(0.0500)	(0.121)	(0.0595)
85%	0.151***	0.166***	0.157***	0.170***	0.165***	0.184***	0.169***	0.145***	0.410**	0.182***
	(0.0441)	(0.0481)	(0.0472)	(0.0515)	(0.0513)	(0.0614)	(0.0650)	(0.0474)	(0.187)	(0.0640)
95%	0.0836**	0.103**	0.100**	0.120**	0.115**	0.149**	0.194**	0.0730	0.0957	0.136*
	(0.0415)	(0.0503)	(0.0494)	(0.0532)	(0.0531)	(0.0665)	(0.0758)	(0.0477)	(0.177)	(0.0698)
Socio‐demographic controls										
Age			0.00311***	0.00363***	0.00360***	0.00362***	0.00327**	0.00400***	0.00450***	0.00305**
			(0.000973)	(0.00105)	(0.00105)	(0.00105)	(0.00134)	(0.00138)	(0.00154)	(0.00123)
Gender			−0.0747***	−0.0452*	−0.0498**	−0.0506**	−0.0398	−0.0311	−0.0140	−0.0461*
			(0.0224)	(0.0237)	(0.0237)	(0.0237)	(0.0297)	(0.0321)	(0.0354)	(0.0275)
Asian			0.203***	0.209***	0.206***	0.207***	0.219***	0.209**	0.135	0.236***
			(0.0596)	(0.0596)	(0.0591)	(0.0595)	(0.0699)	(0.0914)	(0.0824)	(0.0720)
Black			0.276***	0.264***	0.259***	0.252**	0.258**	0.233	0.189	0.266**
			(0.1000)	(0.0978)	(0.0977)	(0.0977)	(0.125)	(0.144)	(0.149)	(0.122)
Other			−0.0373	−0.00466	−0.00687	−0.00795	−0.0749	0.0998	−0.118	0.0361
			(0.0631)	(0.0715)	(0.0713)	(0.0711)	(0.0781)	(0.118)	(0.0792)	(0.0865)
Education			0.0384***	0.0268*	0.0245	0.0258*	0.0147	0.0427**	0.00896	0.0359**
			(0.0145)	(0.0151)	(0.0151)	(0.0151)	(0.0191)	(0.0203)	(0.0223)	(0.0178)
Employment			0.0504	0.0472	0.0470	0.0487	0.0342	0.0449	0.0485	0.0318
			(0.0424)	(0.0433)	(0.0433)	(0.0433)	(0.0557)	(0.0543)	(0.0668)	(0.0499)
Vaccination history & behavioral attitudes controls										
Past vax				0.00789	−0.00436	−0.00443	−0.00426	0.0178	−0.0330	0.0237
				(0.0277)	(0.0280)	(0.0281)	(0.0341)	(0.0387)	(0.0422)	(0.0327)
Individual benefit				0.00456***	0.00424***	0.00428***	0.00486***	0.00362***	0.00234**	0.00526***
				(0.000678)	(0.000691)	(0.000689)	(0.000935)	(0.000805)	(0.00110)	(0.000774)
Prosocial benefit				−0.00158**	−0.00173**	−0.00171**	−0.00163*	−0.00116	−0.000600	−0.00145*
				(0.000732)	(0.000724)	(0.000725)	(0.000897)	(0.00108)	(0.00121)	(0.000834)
Spillovers				0.00197***	0.00173**	0.00179**	0.00226***	0.00117	0.00327***	0.00105
				(0.000710)	(0.000705)	(0.000702)	(0.000853)	(0.00111)	(0.00114)	(0.000829)
Perception controls										
Perceived risk of infection					0.00161***					
					(0.000462)					
Perceived coverage rate					8.53e‐05					
					(0.000527)					
PRI higher						0.00197**				
						(0.000786)				
PRI lower						−0.00106				
						(0.000746)				
PCR higher						0.000706			0.000288	
						(0.00106)			(0.00109)	
PCR lower						−0.000130				−0.000247
						(0.000715)				(0.000801)
Observations	1365	1365	1365	1216	1216	1216	813	556	483	886

*Note*: Standard errors in parentheses; ***p < 0.01, **p < 0.05, *p < 0.1; Participants from the first pilot study (*N* = 149) are excluded from models 4‐6 as data on their behavioral attitudes and social perceptions were not collected, leaving the remaining 1216 participants for analysis; Models are as follows–M1: average treatment effect (ATE), M2: treatment group dummies (TG), M3: M2 + socio‐demographic controls (SDC), M4: M3 + vaccination history & behavioral attitudes controls (VBC), M5: M4 + Perceived Risk of Infection + Perceived Coverage Rate (PER), M6: M4 + PRI Higher/Lower + PCR Higher/Lower (DIST), M7: M4 stratified by high PRI, M8: M4 stratified by low PRI, M9: M4 stratified by high PCR, M10: M4 stratified by low PCR.

Abbreviations: PCR, perceived coverage rate; PRI, perceived risk of infection.

These effects are robust to the introduction of control variables accounting for the individual perceptions of the risk of infection and of the coverage rate (M5). Both underlying perception variables (i.e. *Perceived Risk of Infection* and *Perceived Coverage Rate*) have positive and significant coefficients, suggesting higher stated likelihood of vaccination with greater perceived risk or perceived coverage rate (M5).

When controlling for the difference between perceptions and the treatment coverage rates (*PRI Higher/Lower, PCR Higher*/*Lower*), only treatments at the 65% coverage rate and above have significant effects on map interest (Table 4 – model 6). On average, higher treatment coverage rates lead to greater average likelihood of looking at the map compared to the control group up to the 75% coverage rate, indicating a bandwagoning effect. Results in M6 show that higher rates (85% and 95%) lead to lower average likelihood of looking at the map compared to the 75% treatment, although still significantly greater than the control. Individuals treated with the 65% intervention are 14.2 percentage points (*p* = 0.01) likelier to look at the map compared to the control, 21.6 percentage points (*p* < 0.01) likelier for the 75% treatment, but only 18.4 percentage points (*p* < 0.01) likelier for the 85% treatment, and 14.9 percentage points (*p* = 0.03) likelier for the 95% treatment. There are significant differences in map interest between the 65% (*M* = 0.25, SD = 0.03) and 75% (*M* = 0.33, SD = 0.04) treatment groups (t(349) = −1.8, *p* = 0.08). *Perceived Risk of Infection* has a significant and positive effect on stated vaccination intention (M5). Moreover, the significantly positive coefficient for *PRI Higher* of 0.19 percentage points (*p* = 0.01) further confirms that individuals whose perceived risk of infection is greater than their treatment have higher likelihood of looking at the map compared to the control and those whose treatment equals their perceived risk of infection (M6).

The estimates in M7 and M8 provide evidence that partly supports hypothesis H3 for looking at the map as well: the coefficients for the coverage rate treatments are consistently higher for the participants with higher perceived risk of infection than for the respondents with lower risk of infection.

When we stratify the participants into two groups with high and low perceived coverage rates in M9 and M10, respectively, the estimated coefficients of the control variables *PCR Higher* and *PCR Lower* are positive and negative, respectively, but are not statistically significant, providing no support for hypothesis H4. Finally, results also support H5 with most of the estimated coefficients of the coverage rates larger for those with a high PCR than for those with a low PCR for sufficiently high manipulated coverage rates.

The varying treatment vaccination coverage rates have similar effects on time spent looking at the map and on map interest (Table 5), with findings supporting H1 also for these outcomes, suggesting the same interplay between bandwagoning and free‐riding effects described above. In M2–M5, all treatments have significantly positive effects on time spent looking at the map. When controlling for the difference between perceptions and the treatment coverage rates (*PRI Higher/Lower, PCR Higher*/*Lower*) (M6), however, only messages at the 65% threshold and above have a significant impact on time spent looking at the map compared to the control. In all models, starting from the 65% treatment, participants have longer viewing times at higher coverage rates up to the 75%, pointing toward a bandwagoning effect. Beyond this point, although still significantly greater than the control, participants treated with the 85% and 95% coverage rates spend less time viewing the map than the 75% group, indicating free‐riding balancing out the bandwagoning effects. This is demonstrated in our findings of a 51 percentage points (*p* < 0.01) increase in the time spent looking at the map due to the 65% treatment, a 77 percentage points (*p* < 0.01) increase due to the 75% treatment, but then only a 66 percentage points (*p* < 0.01) increase due to the 85% treatment, and a 52 percentage points (*p* = 0.03) increase due to the 95% treatment, all compared to the control. We find significant differences in time spent looking at the map between the 65% (*M* = 5.8, SD = 1) and 75% (*M* = 10.2, SD = 1.6) groups (t(349) = −2.5, *p* = 0.01) – and the 85% (*M* = 9.6, SD = 2.5) and 95% (*M* = 5.1, SD = 1) groups (t (303) = 1.7, *p* = 0.09). Participants with greater perceived risk of infection spend significantly longer time looking at the map (M5). Specifically, participants whose perceived risk of infection is greater than their treatment spend significantly longer time looking at the map, as indicated by the positive coefficient for *PRI Higher* of 0.67 percentage points (*p* = 0.01) (M6).

**TABLE 5:** Average marginal effects from a double hurdle model of the impact of social norms messaging on time spent looking at a map of influenza vaccine locations (Maptime)

Variables	M1 ATE	M2 TG	M3 SDC	M4 VBC	M5 PER	M6 DIST	M7 High PRI	M8 Low PRI	M9 High PCR	M10 Low PCR
Social norm message treatment										
10%	4.316	0.365**	0.364**	0.458**	0.472***	−0.00728	0.457**	0.488	0.422*	−0.0336
	(4.691)	(0.157)	(0.160)	(0.181)	(0.182)	(0.261)	(0.183)	(0.647)	(0.230)	(0.581)
25%	7.857*	0.412***	0.402***	0.481***	0.489***	0.144	0.479***	0.529	0.361	0.540**
	(4.696)	(0.146)	(0.149)	(0.180)	(0.181)	(0.226)	(0.184)	(0.424)	(0.224)	(0.242)
50%	15.12***	0.314**	0.321**	0.412**	0.423**	0.268	0.543***	−0.336	0.639**	0.291
	(4.619)	(0.159)	(0.162)	(0.183)	(0.185)	(0.201)	(0.192)	(0.393)	(0.257)	(0.221)
65%	5.227	0.484***	0.467***	0.564***	0.566***	0.514**	0.589***	0.515**	−0.266	0.660***
	(4.137)	(0.155)	(0.158)	(0.179)	(0.181)	(0.202)	(0.198)	(0.241)	(0.444)	(0.214)
75%	12.19***	0.529***	0.556***	0.754***	0.767***	0.774***	0.848***	0.679***	1.018**	0.783***
	(4.549)	(0.144)	(0.148)	(0.177)	(0.178)	(0.205)	(0.205)	(0.219)	(0.498)	(0.217)
85%	14.60*	0.532***	0.527***	0.603***	0.590***	0.658***	0.564**	0.637***	1.646**	0.661***
	(8.117)	(0.154)	(0.157)	(0.179)	(0.180)	(0.218)	(0.222)	(0.206)	(0.780)	(0.232)
95%	4.212	0.322**	0.335**	0.409**	0.396**	0.517**	0.661**	0.295	0.384	0.476*
	(4.311)	(0.158)	(0.161)	(0.182)	(0.184)	(0.236)	(0.260)	(0.202)	(0.723)	(0.253)
Socio‐demographic controls										
Age			0.0107***	0.0129***	0.0130***	0.0130***	0.0112**	0.0170***	0.0177***	0.0109**
			(0.00333)	(0.00378)	(0.00380)	(0.00380)	(0.00462)	(0.00597)	(0.00626)	(0.00451)
Gender			−0.254***	−0.163*	−0.181**	−0.181**	−0.133	−0.150	−0.0462	−0.176*
			(0.0768)	(0.0845)	(0.0855)	(0.0854)	(0.103)	(0.137)	(0.142)	(0.101)
Asian			0.600***	0.644***	0.643***	0.646***	0.660***	0.737***	0.466*	0.738***
			(0.159)	(0.175)	(0.177)	(0.177)	(0.204)	(0.284)	(0.272)	(0.213)
Black			0.791***	0.814***	0.818***	0.793***	0.778**	0.839*	0.676	0.840**
			(0.261)	(0.278)	(0.281)	(0.280)	(0.360)	(0.432)	(0.450)	(0.349)
Other			−0.128	−0.0218	−0.0200	−0.0237	−0.318	0.421	−0.661	0.125
			(0.238)	(0.261)	(0.263)	(0.262)	(0.342)	(0.396)	(0.591)	(0.298)
Education			0.129***	0.0917*	0.0857	0.0887*	0.0438	0.188**	0.0390	0.133**
			(0.0486)	(0.0534)	(0.0540)	(0.0539)	(0.0652)	(0.0862)	(0.0888)	(0.0646)
Employment			0.180	0.150	0.149	0.153	0.119	0.147	0.188	0.0910
			(0.143)	(0.152)	(0.153)	(0.153)	(0.191)	(0.226)	(0.263)	(0.179)
Vaccination history & behavioral attitudes controls										
Past vax				0.0286	−0.0176	−0.0165	−0.0153	0.0685	−0.140	0.0900
				(0.100)	(0.102)	(0.103)	(0.119)	(0.169)	(0.171)	(0.121)
Individual benefit				0.0157***	0.0148***	0.0149***	0.0161***	0.0157***	0.0101**	0.0188***
				(0.00241)	(0.00247)	(0.00246)	(0.00321)	(0.00350)	(0.00421)	(0.00289)
Prosocial benefit				−0.00551**	−0.00618**	−0.00611**	−0.00543*	−0.00498	−0.00298	−0.00523*
				(0.00264)	(0.00267)	(0.00266)	(0.00312)	(0.00472)	(0.00479)	(0.00312)
Spillovers				0.00698***	0.00617**	0.00645**	0.00782***	0.00472	0.0131***	0.00370
				(0.00256)	(0.00258)	(0.00257)	(0.00296)	(0.00483)	(0.00460)	(0.00306)
Perception controls										
Perceived risk of infection					0.00563***					
					(0.00166)					
Perceived coverage rate					0.000287					
					(0.00190)					
PRI higher						0.00672**				
						(0.00279)				
PRI lower						−0.00380				
						(0.00263)				
PCR higher						0.00268			0.00153	
						(0.00382)			(0.00443)	
PCR lower						−0.000404				−0.000849
						(0.00260)				(0.00297)
Observations	325^a^	1365	1365	1216	1216	1216	813	556	483	886

*Note*: Standard errors in parentheses; ***p < 0.01, **p < 0.05, *p < 0.1;

^a^ Average treatment effect is calculated only for those who choose to view the map (*N* = 325); Participants from the first pilot study (*N* = 149) are excluded from models 4‐6 as data on their behavioral attitudes and social perceptions were not collected, leaving the remaining 1216 participants for analysis; Models are as follows–M1: average treatment effect (ATE), M2: treatment group dummies (TG), M3: M2 + socio‐demographic controls (SDC), M4: M3 + vaccination history & behavioral attitudes controls (VBC), M5: M4 + Perceived Risk of Infection + Perceived Coverage Rate (PER), M6: M4 + PRI Higher/Lower + PCR Higher/Lower (DIST), M7: M4 stratified by high PRI, M8: M4 stratified by low PRI, M9: M4 stratified by high PCR, M10: M4 stratified by low PCR.

Abbreviations: PCR, perceived coverage rate; PRI, perceived risk of infection.

Overall, the effects of perceived risk of infection and perceived coverage rates on interest in and duration of looking at the map are similar to those for self‐reported vaccination intention, although only the coefficients for *PRI Higher* are significant.

In the stratified double hurdle models for the time spent looking at the map, the estimates in M7‐M8 support H3 for sufficiently high manipulated coverage rates. In M9‐M10 the sign of estimates of *PCR Higher* and *PCR Lower* are aligned with H4, although the effects are not statistically significant, though this could be due to the reduced sample size in these stratified regressions. Finally there is also some evidence in support of H5 with the coefficients of the manipulated coverage rates, when significant, higher for those with a high PCR than for those with low PCR. Therefore, also for this outcome variable, results highlight, the significant heterogeneity of the treatment effects when these are interacted with either the different perceptions of the risk of infection or with the different perceptions of the coverage rates.

For the probability of downloading the calendar reminder, although treatment effect coefficients are comparable in size and sign to those of the other behavioral outcomes, these tend to be non significant in most specifications, probably due to the small number of subjects (*N* = 133) that chose to download the reminder. The exception is for the 85% and 95% manipulated vaccination coverage rates in M6 (i.e. model with controls for the difference between perceptions and treatment coverage rates, *PRI Higher/Lower, PCR Higher*/*Lower*) that have a significant and positive effect (Table 6 – model 6). Coefficients for treatment groups below and above the 85% coverage rate are positive but lower, suggesting a peak of 9.2 percentage points (*p* = 0.03) higher probability of calendar reminder download at the 85% treatment. This is consistent with findings for the map‐related vaccination intention measures–a bandwagoning effect potentially overweighted by a free‐riding effect above a certain population coverage rate. Although those treated with the 65% and 75% coverage rates are 4.5 percentage points (*p* = 0.27) and 4.4 percentage points (*p* = 0.30) more likely to download the calendar reminder than the control, these probabilities are lower compared to those treated with the 85% rate. Similarly, individuals given the higher treatment of 95% are 7.8 percentage points (*p* = 0.09) more likely to download the calendar reminder compared to the control, but this effect is lower than the 85% group. There are no significant differences between treatment groups for calendar download likelihood. Perceptions variables have the same signs as in the regressions for the other behavioral proxies.

**TABLE 6:** Average marginal effects from a logit regression of the impact of social norms messaging on the probability of downloading a calendar reminder to get the influenza vaccine at the start of the following flu season (Cal)

Variables	M1	M2	M3	M4	M5	M6
	ATE	TG	SDC	VBC	PER	DIST
Social norm message treatment						
10%	0.0582*	0.0653*	0.0585*	0.0448	0.0461	−0.0459
	(0.0309)	(0.0342)	(0.0339)	(0.0363)	(0.0360)	(0.0557)
25%	0.0239	0.0319	0.0301	0.00792	0.00623	−0.0652
	(0.0255)	(0.0342)	(0.0338)	(0.0384)	(0.0381)	(0.0505)
50%	0.0539*	0.0617*	0.0590*	0.0482	0.0495	0.0241
	(0.0308)	(0.0346)	(0.0341)	(0.0365)	(0.0361)	(0.0407)
65%	0.0539*	0.0617*	0.0557	0.0438	0.0408	0.0451
	(0.0308)	(0.0346)	(0.0341)	(0.0364)	(0.0362)	(0.0404)
75%	0.0301	0.0387	0.0376	0.0217	0.0202	0.0439
	(0.0261)	(0.0339)	(0.0335)	(0.0378)	(0.0376)	(0.0424)
85%	0.0722**	0.0765**	0.0720**	0.0560	0.0503	0.0924**
	(0.0321)	(0.0337)	(0.0333)	(0.0358)	(0.0353)	(0.0423)
95%	0.0321	0.0408	0.0382	0.0233	0.0167	0.0783*
	(0.0286)	(0.0358)	(0.0355)	(0.0378)	(0.0375)	(0.0468)
Socio‐demographic controls						
Age			0.00215***	0.00231***	0.00228***	0.00233***
			(0.000702)	(0.000773)	(0.000766)	(0.000768)
Gender			−0.0151	−0.00211	−0.00628	−0.00679
			(0.0160)	(0.0171)	(0.0171)	(0.0170)
Asian			0.114**	0.102**	0.0979**	0.103**
			(0.0500)	(0.0508)	(0.0493)	(0.0504)
Black			0.181**	0.210**	0.201**	0.195**
			(0.0915)	(0.0949)	(0.0913)	(0.0909)
Other			0.134**	0.176**	0.177**	0.173**
			(0.0672)	(0.0753)	(0.0743)	(0.0736)
Education			0.00457	0.000900	−0.000343	0.00107
			(0.0101)	(0.0107)	(0.0107)	(0.0107)
Employment			0.0355	0.0324	0.0335	0.0373
			(0.0307)	(0.0320)	(0.0321)	(0.0323)
Vaccination history & behavioral attitudes controls						
Past vax				0.0152	0.00505	0.00350
				(0.0192)	(0.0192)	(0.0193)
Individual benefit				0.00252***	0.00216***	0.00223***
				(0.000540)	(0.000542)	(0.000542)
Prosocial benefit				−0.000370	−0.000446	−0.000448
				(0.000506)	(0.000491)	(0.000494)
Spillovers				0.000421	0.000206	0.000289
				(0.000487)	(0.000473)	(0.000475)
Perception controls						
Perceived risk of infection					0.00153***	
					(0.000382)	
Perceived coverage rate					0.000143	
					(0.000369)	
PRI higher						0.00161***
						(0.000600)
PRI lower						−0.00117*
						(0.000663)
PCR higher						−0.000261
						(0.000749)
PCR lower						−0.000601
						(0.000514)
Observations	1365	1365	1365	1216	1216	1216

*Note*: Standard errors in parentheses; ***p < 0.01, **p < 0.05, *p < 0.1; Participants from the first pilot study (*N* = 149) are excluded from models 4‐6 as data on their behavioral attitudes and social perceptions were not collected, leaving the remaining 1216 participants for analysis; Models are as follows–M1: average treatment effect (ATE), M2: treatment group dummies (TG), M3: M2 + socio‐demographic controls (SDC), M4: M3 + vaccination history & behavioral attitudes controls (VBC), M5: M4 + Perceived Risk of Infection + Perceived Coverage Rate (PER), M6: M4 + PRI Higher/Lower + PCR Higher/Lower (DIST).

Abbreviations: PCR, perceived coverage rate; PRI, perceived risk of infection.

These findings suggest heterogeneity of the effect of various vaccination coverage rates on self‐reported and behavioral measures for vaccination intention. For behavioral measures, higher treatment coverage rates have higher vaccination intention up to the 75%‐85% threshold, indicating *bandwagoning*. Beyond these treatment intensities, higher treatment coverage rates result in smaller (albeit still positive) vaccination intention, suggesting that a *free‐riding* effect partially crowds out the *bandwagoning* effect. Treatment coverage rates, however, have a monotonically increasing effect on self‐reported vaccination intention when controlling for perceived risk of infection and coverage rates.

To summarize, our empirical results support some of the hypotheses informed by the theoretical framework presented above. In particular, the results provide evidence in support of the interplay between *bandwagoning* and *free‐riding* effects impacting self‐reported and behavioral measures for vaccination intention. Furthermore, these effects differ depending on perceived risk of infection and perceived coverage rates. Our results remain consistent after further extensive robustness checks employing probit models (instead of logit models); using binary (instead of continuous) variables for our perception controls; modeling differently the interaction effects between the treatment effects and the perception variables; stratifying differently the participants, for example, splitting them based on whether the coverage rates treatments are below or above 50% of the population; as well as using a different set of control variables selected via a post double selection least absolute shrinkage and selection operator (LASSO) method (findings available upon request).

## 7 CONCLUSION

This study is arguably the first to experimentally measure the effects of different coverage rates messages on vaccination intentions to better capture the interplay between potential free‐riding and bandwagoning effects. We evaluate vaccination intentions with self‐reported and directly observed behavioral measures, and examine how intentions are moderated by individual perceptions. Perceived coverage rates and perceived risks of infection reflect imperfect information and heterogeneous beliefs on these measures across individuals. They influence how individuals experience different treatment messages and modify the impact of coverage rates messages on influenza vaccination intention.

Our findings suggest that social norms interventions using population vaccination coverage rates increase both self‐reported and behavioral measures for vaccination intentions. However, the magnitude of the effect varies across self‐reported and behavioral measures, as well as with the coverage rates. While our research design does not enable disentangling the roles played by bandwagoning and free‐riding effects, our results suggest that the interplay between *free‐riding* and *bandwagoning* effects differs across treatment intensities and outcome measures.

For self‐reported vaccination intention, we find that groups treated with the information that the vaccination coverage rates in the population are at 65% or above have significantly higher average vaccination intention than the control group. For coverage rates up to 75%, individuals treated with higher coverage rates have greater vaccination intention than those treated with lower rates, consistent with a bandwagoning effect. However, for coverage rates above 75% the treatment effects, albeit still positive, stop increasing and remain flat (or even decline), signaling that, besides a bandwagoning effect, a free‐riding effect may also kick in at high levels of the coverage rate. We also find significant heterogeneity of the treatment effects depending on the invidual perceptions of risks of infection and of the coverage rates, in line with most hypotheses derived from our theoretical framework.

For behavioral measures for vaccination intention, social norms also have a positive impact on vaccination intention. However, the effect increases up to the 75% coverage rate and decreases beyond that level, suggesting that the interplay between *free‐riding* and *bandwagoning* effects varies across social norms intensities. While the positive effect of the norm consistent with a *bandwagoning* effect is found to dominate at higher treatment intensities, its effect is partially crowded out by a *free‐riding* effect at these levels. Also for the behavioral outcome variables we find significant heterogeneity of the treatment effects depending on the invidual perceptions of risks of infection and of the coverage rates.

We also find that individual perceptions moderate the effect of coverage rates treatments on behavioral measures for vaccination intention. Although not all perception variables are statistically significant, in general, individuals who have higher perceived risks of infection or higher perceived coverage rates than their coverage rate treatment have greater vaccination intention than those untreated or whose treatment equals their perceived risks of infection or perceived coverage rates. Conversely, those whose perceived risks of infection or perceived coverage rates are less than their treatment have lower vaccination intention. Additionally, the further their perceptions lie above or below their treatment, the stronger they increase or decrease vaccination intention.

This study has limitations. First, online experiments come with the challenges of ensuring a representative group of respondents or enabling participants playing games of strategic interaction (e.g. Böhm et al., 2016, 2017). We use Prolific, a world‐leading crowd‐sourced platform for research purposes with a participant pool of over 50,000. Peer et al. (2017) demonstrate that Prolific participants provide more truthful and reliable answers compared to Amazon M‐Turk participants (another widely used online experiment platform). Second, our behavioral measures for vaccination intention are only imperfect proxies for actual vaccine uptake, although arguably still closer to real‐world behavior than self‐reported intentions. Further research using data on actual vaccine uptake is warranted. Third, our calendar download behavioral measure did not yield consistently significant findings, possibly because it was elicited after the other measures of vaccination intentions. Counter‐balanced ordering of the different vaccination intention questions in future experiments may yield alternate results. Fourth, while we attempt to cover a wide range of coverage rates, we are ultimately unable to measure the effect of a continuous range of population coverage rates on individual vaccination intention. Additionally, we run a between‐subject experiment whereby each participant is exposed to one coverage rate treatment. We did not investigate how each participant may change their vaccination behavior in response to exposure to various treatment intensities, or to different scenarios about directly manipulated free‐riding or bandwagoning effects. Using a within‐subjects design, further research should investigate how social norms messages of varying intensity affect individual vaccination decision‐making. It is possible that our measure of perceived risk of infection may not accurately capture participants' free‐riding attitudes as participants may not fully understand the indirect benefits of others' vaccination upon which they can free‐ride and not vaccinate. Bohm et al. (2016), for example, give participants quite detailed experimental instructions to make sure they understand how they could profit from others being vaccinated. In our experiment, we did not use such explicit instructions. However, due to the randomization of participants, this potential lack of knowledge would likely be similar across control and treatment groups. This suggests that the lower intent to vaccinate that we observe at higher coverage levels may be at least in part due to some free‐riding effect, even if participants are not fully aware of, or do not understand, that they have a lowered risk of infection. If anything, the magnitude of the free‐riding effect that we observe at higher coverage levels may be downward biased: participants' intent to vaccinate may be even lower if their ability to free‐ride would have been made more salient to them. Further investigation into the interaction between awareness and motivation behind free‐riding at higher coverage levels is warranted. Relatedly, while in our study we purportedly model, elicit, and account for, the individual perceptions about the coverage rate, we do not assess the full distribution of the individual *a priori* beliefs about each possible level of the coverage rates. As a consequence, we cannot exclude the possibility that some participants receiving messages about very low or very high coverage rates (e.g. those allocated to the 10% or 95% coverage rates treatments) could perceive those coverage rates as unrealistically low or unrealistically high, respectively. While the variables on the difference between perceived coverage rates and manipulated coverage rates partly account for these effects, the low differences in treatment effects on vaccination intentions between the control group and the 10% or the 95% coverage rates treatments could be further explained by those participants being less likely to react to their, unrealistic, messages. Further research could also investigate the role that different beliefs about the reproduction number (so called R0) of seasonal flu play on vaccination and on the effectiveness of social norms.

Despite these limitations, this study furthers our current understanding of the effect that social norms messages of different population vaccination coverage rates have on vaccination intention. Our study empirically tests the theories of social norms and prevalence‐elastic demand for prevention in the context of seasonal influenza vaccination. It demonstrates that social norms messages as a public health intervention can be less or more effective, depending on the population coverage rate communicated in the message. In designing policies that leverage on these types of messages to increase vaccination uptake, policymakers should consider the potential interplay between *bandwagoning* and *free‐riding* effects, how this varies across different coverage rates messages, and how it is shaped by heterogeneous individual perceptions. Importantly, our findings suggest that an intervention that conveys the message that 75% of the population are vaccinated increases interest in vaccinations by up to 70% compared to a group that receives no intervention.

Our results further suggest that a 75% message will maximize vaccine uptake. At any higher coverage rates, uptake will still be higher compared to a control group, but the impact will be smaller. Populations with vaccination rates lower than 80% are the prime target for public health interventions because rates are below the threshold required for herd immunity (Plans‐Rubió, 2012). An intervention messaging a 75% social norm targeted at non‐vaccinated individuals would be more impactful than one messaging 80% or higher because free‐riding effects would make the policy less effective. Moreover, due to the moderating effect of perceptions, social norm messages would be more effective for individuals with either very high or very low perceived risk of infection or perceived coverage rates, depending on if their perceptions lies above or below the messaged coverage rate, compared to those with perceptions very close to the messaged coverage rate. Further understanding of the interplay between the vaccination coverage rate, perceived risk of infection, and perceived coverage rate of communities may help policymakers tailor their social norms messaging campaigns in order to more effectively increase vaccine uptake. Areas where the population coverage rate is greater than 75% and/or average perceived risk of infection or perceived coverage rate is very high or low compared to the population coverage rate may benefit from social norms messages. Other communities may benefit from alternative interventions that emphasize past vaccination status or the individual benefit of vaccinating.

There is an increasing interest on the unintended consequences and backfiring (‘spillover’) effects of behavioral policy interventions, including social norms messages and nudges (Allcott & Rogers, 2014; Dolan & Galizzi, 2015; Schubert, 2017; Truelove et al., 2014). At the same time, there is a growing interest in measuring heterogeneous treatment effects and in understanding which individual characteristics and beliefs moderate different responses to behavioral interventions (Angrist, 2004; Imai & Ratkovic, 2013; Schultz et al., 2016). This paper also contributes to these streams of research and their policy implications by looking at the specific case of flu vaccination intention.

## CONFLICT OF INTEREST

We declare that we have no conflicts of interest.

## AUTHOR CONTRIBUTION

The sponsors of the study were not involved in the study design, data collection, data analysis, data interpretation, or writing of the report. All authors had full access to all the data used in this study and had final responsibility for the decision to submit the article for publication.

## Supporting information

Supporting Information 1

## Data Availability

Research data are not shared.

## ENDNOTE

^1^
The stratified regressions have not been run for the calendar reminder variable due to the small number of observations.
