# Supplementary material for: Bandwagoning, free‐riding and heterogeneity in influenza vaccine decisions: An online experiment
Source: Health Econ. 2022 Jan 6;31(4):614–46. doi: 10.1002/hec.4467 (PMC9305895; doi:10.1002/hec.4467)
Supplement: Supplementary file 1 — Supporting Information 1 [file HEC-31-614-s001.docx]

**Appendix for “Bandwagoning, Free-Riding and Heterogeneity in Influenza Vaccine Decisions: An Online Experiment”**

**Figure A. Social norms message for 25% population vaccination coverage level treatment group.**


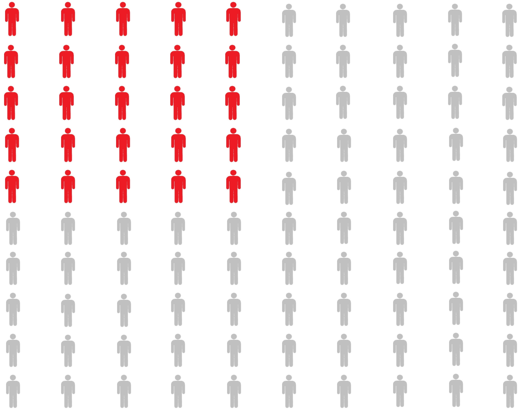


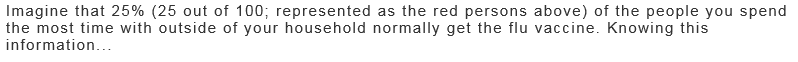


**Figure B. Vaccination intention questions**

**B1. Vax: stated vaccination intention**


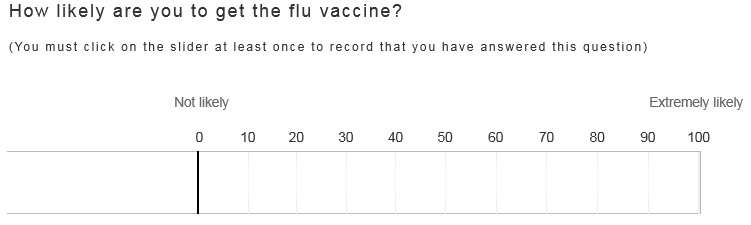


**B2. Map: participant’s interest in looking at the map**


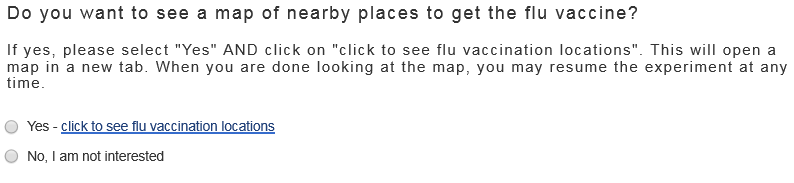


**B3. Maptime: time participant spent looking at the map (recorded by Qualtrics and not observed by the participant)**


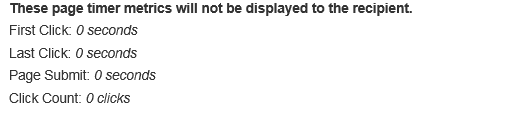


**B4. Cal: participant’s interest in downloading the calendar reminder**


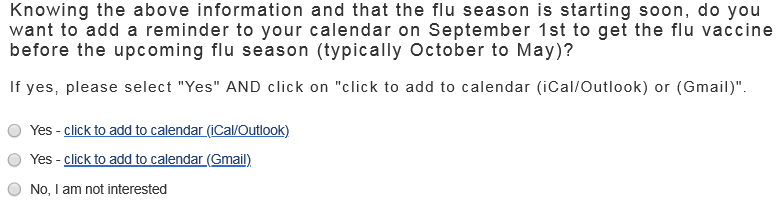


**C: Log-likelihood functions**

The following variables are used in all of our log-likelihood functions: *T_ik_* is a vector of our seven treatment variables taking the value of 1 if participant *i* is treated with the specified social norm *k*. For the control group, *T_ik_* = 0, $\forall i$. Vector *P_i_* contains the perception variables, *Perceived Risk of Infection (PRI) Higher*, *PRI Lower*, *Perceived Coverage Rate (PCR) Higher*, and *PCR Lower.* Vector *X_i_* contains the socio-demographic (*Age, Gender, Ethnicity, Education, Employment*) and vaccination history and behavioral attitude (*Past Vax, Individual Benefit, Prosocial Benefit, Spillovers*) control variables. Coefficients of *T_ik_, P_i_*, and *X_i_* are stored in vectors β, γ, and δ, respectively.

**C1: Logit model to measure the impact of social norm messaging on interest in viewing the online map of nearby flu vaccine pharmacies (*Map_ik_*).**

$$\ln L(\beta,\gamma,\delta;{Map}_{ik}, T_{ik}, X_{i},P_{i}) = \sum_{i=1}^{n} \left[ \begin{aligned} {Map}_{i}\ln F\left( {\beta_{k}T}_{ik}+\gamma P_{i} +\delta X_{i} \right)+ \\ \left( 1-{Map}_{i} \right)\ln\left( 1-F\left( {\beta_{k}T}_{ik}+\gamma P_{i} +\delta X_{i} \right) \right) \end{aligned} \right]$$

where $F\left( z \right)=\frac{e^{z}}{1+e^{z}}$.

**C2: Cragg’s Double Hurdle Model to measure the impact of social norm messaging on time spent viewing the online map of nearby flu vaccine pharmacies (*Maptime_ik_*).**

$lnL=\sum_{0} ln\left[ 1-\Phi\left( {\omega D}_{i} \right)\Phi\left( \frac{{\omega D}_{i}}{\sigma_{i}} \right) \right]+\sum_{+} ln\left[ \Phi\left( \omega D_{i} \right)\frac{1}{\sigma_{i}}\varphi\left( \frac{y_{i}-{\omega D}_{i}}{\sigma_{i}} \right) \right]$

where $\Phi(\cdot)$ and $\varphi(\cdot)$ are the standard normal cumulative distribution and the probability density functions, respectively, and ${\omega D}_{ik}={\beta_{k}T}_{ik}+\gamma P_{i} +{\delta X}_{i}$.

**C3: Logit model to measure the impact of social norm messaging on interest in downloading the calendar reminder to vaccinate at the start of the next flu season (*Cal_ik_*).**

$$\ln L(\beta,\gamma,\delta;{Cal}_{ik}, T_{ik}, X_{i},P_{i}) = \sum_{i=1}^{n} \left[ \begin{aligned} {Cal}_{i}\ln F\left( {\beta_{k}T}_{ik}+\gamma P_{i} +\delta X_{i} \right)+ \\ \left( 1-{Cal}_{i} \right)\ln\left( 1-F\left( {\beta_{k}T}_{ik}+\gamma P_{i} +\delta X_{i} \right) \right) \end{aligned} \right]$$

where $F\left( z \right)=\frac{e^{z}}{1+e^{z}}$.

| **Table D. Kruskal-Wallis Tests** | | | |
| --- | --- | --- | --- |
| **Variables** | **X^2^** | **p** |  |
| **Outcome Measures** |  |  |  |
| Vax | 22.21 | < 0.01 |  |
| Map | 9.64 | 0.21 |  |
| Maptime | 15.57 | 0.03 |  |
| Cal | 7.57 | 0.37 |  |
| **Controls** |  |  |  |
| **Socio-demographic characteristics** |  |  |  |
| Age | 12.76 | 0.08 |  |
| Gender | 0.15 | 1 |  |
| Ethnicity | 0.68 | 0.999 |  |
| Education | 2.99 | 0.89 |  |
| Employment | 1.11 | 0.99 |  |
| **Vaccination history & other behavioral attitudes** |  |  |  |
| Past Vax | 1.11 | 0.99 |  |
| Individual Benefit | 1.53 | 0.98 |  |
| Prosocial Benefit | 7.87 | 0.35 |  |
| Spillovers | 6.99 | 0.43 |  |
| **Perception** |  |  |  |
| Perceived Risk of Infection (PRI) | 11.4 | 0.12 |  |
| Perceived Coverage Rate (PCR) | 5.3 | 0.63 |  |
| PRI Higher | 910.45 | < 0.01 |  |
| PRI Lower | 807.66 | < 0.01 |  |
| PCR Higher | 699.63 | < 0.01 |  |
| PCR Lower | 872.73 | < 0.01 |  |
| *Note: Kruskal-Wallis tests determine significant differences between any of the control and treatment groups* | | | |

| **Table E. Mann Whitney Tests** | | | | | |
| --- | --- | --- | --- | --- | --- |
| **Variables** | **Group A** | **Group B** | **Z-statistic** | **P-value** | **M_B_-M_A_** |
| **Outcome Measures** |  |  |  |  |  |
| Vax | control | All Treated | -2.48 | 0.01 | 6.37 |
|  |  | 10% | -0.54 | 0.59 | 0.58 |
|  |  | 25% | -0.87 | 0.38 | 1.79 |
|  |  | 50% | -0.94 | 0.35 | 3.03 |
|  |  | 65% | -3.04 | <0.01 | 10.19 |
|  |  | 75% | -2.51 | 0.01 | 9.80 |
|  |  | 85% | -2.55 | 0.01 | 10.07 |
|  |  | 95% | -2.21 | 0.03 | 9.10 |
| Map | control | All Treated | -3.60 | <0.01 | 0.12 |
|  |  | 10% | -2.29 | 0.02 | 0.09 |
|  |  | 25% | -3.01 | <0.01 | 0.13 |
|  |  | 50% | -1.99 | 0.05 | 0.08 |
|  |  | 65% | -2.71 | 0.01 | 0.11 |
|  |  | 75% | -4.31 | <0.01 | 0.19 |
|  |  | 85% | -3.49 | <0.01 | 0.15 |
|  |  | 95% | -2.05 | 0.04 | 0.08 |
| Maptime | control | All Treated | -3.84 | <0.01 | 4.61 |
|  |  | 10% | -2.38 | 0.02 | 2.61 |
|  |  | 25% | -3.18 | <0.01 | 4.46 |
|  |  | 50% | -2.34 | 0.02 | 4.84 |
|  |  | 65% | -2.85 | <0.01 | 3.26 |
|  |  | 75% | -4.67 | <0.01 | 7.61 |
|  |  | 85% | -3.74 | <0.01 | 7.03 |
|  |  | 95% | -2.18 | 0.03 | 2.49 |
| Cal | control | All Treated | -1.97 | 0.05 | 0.04 |
|  |  | 10% | -1.78 | 0.08 | 0.05 |
|  |  | 25% | -0.91 | 0.36 | 0.02 |
|  |  | 50% | -1.82 | 0.07 | 0.05 |
|  |  | 65% | -1.66 | 0.10 | 0.05 |
|  |  | 75% | -1.18 | 0.24 | 0.03 |
|  |  | 85% | -2.34 | 0.02 | 0.07 |
|  |  | 95% | -1.15 | 0.25 | 0.03 |
| **Controls** |  |  |  |  |  |
| **Socio-demographic characteristics** |  |  |  |  |  |
| Age | control | All Treated | -2.53 | 0.01 | 1.94 |
|  |  | 10% | -0.38 | 0.71 | -0.28 |
|  |  | 25% | -3.02 | 0.00 | 3.00 |
|  |  | 50% | -1.79 | 0.07 | 1.67 |
|  |  | 65% | -0.71 | 0.48 | 0.96 |
|  |  | 75% | -2.44 | 0.01 | 2.45 |
|  |  | 85% | -3.27 | 0.00 | 3.76 |
|  |  | 95% | -1.88 | 0.06 | 2.03 |
| Gender | control | All Treated | -0.05 | 0.96 | 0.00 |
|  |  | 10% | <0.0001 | 1.00 | 0.00 |
|  |  | 25% | 0.08 | 0.94 | -0.01 |
|  |  | 50% | 0.08 | 0.94 | -0.01 |
|  |  | 65% | -0.35 | 0.73 | 0.02 |
|  |  | 75% | 0.02 | 0.99 | 0.00 |
|  |  | 85% | 0.08 | 0.94 | -0.01 |
|  |  | 95% | -0.11 | 0.91 | 0.00 |
| Ethnicity | control | All Treated | -0.16 | 0.88 | 0.02 |
|  |  | 10% | -0.79 | 0.43 | 0.08 |
|  |  | 25% | -0.35 | 0.73 | 0.01 |
|  |  | 50% | -0.09 | 0.93 | 0.02 |
|  |  | 65% | -0.43 | 0.67 | 0.02 |
|  |  | 75% | 0.08 | 0.94 | 0.04 |
|  |  | 85% | 0.56 | 0.58 | -0.02 |
|  |  | 95% | 0.31 | 0.76 | 0.02 |
| Education | control | All Treated | -0.57 | 0.57 | 0.06 |
|  |  | 10% | 0.26 | 0.79 | -0.01 |
|  |  | 25% | -1.44 | 0.15 | 0.15 |
|  |  | 50% | -0.05 | 0.96 | 0.03 |
|  |  | 65% | -0.91 | 0.36 | 0.09 |
|  |  | 75% | -0.94 | 0.35 | 0.10 |
|  |  | 85% | 0.04 | 0.97 | 0.03 |
|  |  | 95% | -0.02 | 0.99 | 0.01 |
| Employment | control | All Treated | -0.79 | 0.43 | 0.02 |
|  |  | 10% | -1.64 | 0.10 | 0.05 |
|  |  | 25% | -0.39 | 0.69 | 0.01 |
|  |  | 50% | -0.51 | 0.61 | 0.02 |
|  |  | 65% | 0.06 | 0.95 | 0.00 |
|  |  | 75% | -1.15 | 0.25 | 0.04 |
|  |  | 85% | 0.02 | 0.98 | 0.00 |
|  |  | 95% | -0.57 | 0.57 | 0.02 |
| **Vaccination history & other behavioral attitudes** |  |  |  |  |  |
| Past Vax | control | All Treated | 0.51 | 0.61 | -0.01 |
|  |  | 10% | 0.38 | 0.70 | -0.01 |
|  |  | 25% | 0.27 | 0.79 | -0.01 |
|  |  | 50% | -0.01 | 0.99 | 0.00 |
|  |  | 65% | 1.43 | 0.15 | -0.05 |
|  |  | 75% | 0.02 | 0.98 | 0.00 |
|  |  | 85% | -0.09 | 0.93 | 0.01 |
|  |  | 95% | 0.38 | 0.70 | -0.01 |
| Individual Benefit | control | All Treated | -0.73 | 0.46 | 2.53 |
|  |  | 10% | -0.85 | 0.40 | 3.54 |
|  |  | 25% | -0.78 | 0.43 | 3.24 |
|  |  | 50% | -0.21 | 0.83 | 0.25 |
|  |  | 65% | -0.30 | 0.76 | 2.35 |
|  |  | 75% | -0.84 | 0.40 | 3.88 |
|  |  | 85% | -0.42 | 0.67 | 2.09 |
|  |  | 95% | -0.45 | 0.65 | 2.30 |
| Prosocial Benefit | control | All Treated | -1.93 | 0.05 | 5.27 |
|  |  | 10% | -1.65 | 0.10 | 6.16 |
|  |  | 25% | -2.07 | 0.04 | 7.70 |
|  |  | 50% | -1.15 | 0.25 | 4.46 |
|  |  | 65% | -0.73 | 0.47 | 2.64 |
|  |  | 75% | -0.74 | 0.46 | 2.26 |
|  |  | 85% | -2.15 | 0.03 | 7.53 |
|  |  | 95% | -1.67 | 0.09 | 6.00 |
| Spillovers | control | All Treated | -0.98 | 0.33 | 3.04 |
|  |  | 10% | -0.45 | 0.65 | 2.13 |
|  |  | 25% | -1.40 | 0.16 | 5.54 |
|  |  | 50% | -0.82 | 0.41 | 3.36 |
|  |  | 65% | 0.26 | 0.79 | -0.31 |
|  |  | 75% | -0.08 | 0.93 | 0.33 |
|  |  | 85% | -1.61 | 0.11 | 6.03 |
|  |  | 95% | -1.08 | 0.28 | 4.07 |
| **Perception** |  |  |  |  |  |
| Perceived Risk of Infection | control | All Treated | -1.93 | 0.05 | 4.85 |
|  |  | 10% | -0.83 | 0.41 | 2.81 |
|  |  | 25% | -1.50 | 0.13 | 4.80 |
|  |  | 50% | -0.34 | 0.74 | 1.71 |
|  |  | 65% | -1.53 | 0.13 | 5.10 |
|  |  | 75% | -1.20 | 0.23 | 3.69 |
|  |  | 85% | -2.81 | < 0.01 | 9.36 |
|  |  | 95% | -1.97 | 0.05 | 6.41 |
| Perceived Coverage Rate | control | All Treated | 0.96 | 0.34 | -2.38 |
|  |  | 10% | 0.33 | 0.74 | -1.53 |
|  |  | 25% | 1.37 | 0.17 | -4.05 |
|  |  | 50% | 0.27 | 0.79 | -1.13 |
|  |  | 65% | 1.45 | 0.15 | -4.70 |
|  |  | 75% | 0.97 | 0.33 | -2.63 |
|  |  | 85% | 0.82 | 0.42 | -2.52 |
|  |  | 95% | -0.12 | 0.91 | -0.14 |
| PRI Higher | control ^a^ | Treated (perceived infection risk > treatment) | -32.07 | <0.01 | 34.07 |
| PRI Lower | control ^a^ | Treated (perceived infection risk < treatment) | -33.94 | <0.01 | 33.67 |
| PCR Higher | control ^a^ | Treated (perceived coverage rate > treatment) | -34.34 | <0.01 | 24.75 |
| PCR Lower | control ^a^ | Treated (perceived coverage rate < treatment) | -30.52 | <0.01 | 40.15 |
| *Note: Mann Whitney tests determine significant differences between the control and each of the treatment groups; ^a^ Control groups for perception variables include participants in the actual control group and those whose perception equaled their treatment.* | | | | | |
